# Surf4 (Erv29p) binds amino-terminal tripeptide motifs of soluble cargo proteins with different affinities, enabling prioritization of their exit from the endoplasmic reticulum

**DOI:** 10.1371/journal.pbio.2005140

**Published:** 2018-08-07

**Authors:** Ying Yin, Mekka R. Garcia, Alexander J. Novak, Allison M. Saunders, Raira S. Ank, Anna S. Nam, Larry W. Fisher

**Affiliations:** Matrix Biochemistry Section, National Institute of Dental and Craniofacial Research, National Institutes of Health, Bethesda, Maryland, United States of America; Weizmann Institute of Science, Israel

## Abstract

Some secreted proteins that assemble into large complexes, such as extracellular matrices or hormones and enzymes in storage granules, must be kept at subaggregation concentrations during intracellular trafficking. We show surfeit locus protein 4 (Surf4) is the cargo receptor that establishes different steady-state concentrations for a variety of soluble cargo proteins within the endoplasmic reticulum (ER) through interaction with the amino-terminal tripeptides exposed after removal of leader sequences. We call this motif the ER-Exit by Soluble Cargo using Amino-terminal Peptide-Encoding motif (ER-ESCAPE motif). Proteins that most readily aggregate in the ER lumen (e.g., dentin sialophosphoprotein [DSPP] and amelogenin, X-linked [AMELX]) have strong ER-ESCAPE motifs to inhibit aggregate formation, while less susceptible cargo exhibits weaker motifs. Specific changes in a single amino acid of the tripeptide result in aggregate formation and failure to efficiently traffic cargo out of the ER. A logical subset of 8,000 possible tripeptides starting a model soluble cargo protein (growth hormone) established a continuum of steady-state ER concentrations ranging from low (i.e., high affinity for receptor) to the highest concentrations associated with bulk flow–limited trafficking observed for nonbinding motifs. Human cells lacking *Surf4* no longer preferentially trafficked cargo expressing strong ER-ESCAPE motifs. Reexpression of Surf4 or expression of yeast’s ortholog, ER-derived vesicles protein 29 (Erv29p), rescued enhanced ER trafficking in *Surf4*-null cells. Hence our work describes a new way of preferentially exporting soluble cargo out of the ER that maintains proteins below the concentrations at which they form damaging aggregates.

## Author summary

The majority of proteins secreted from eukaryotic cells are targeted to the endoplasmic reticulum (ER) for folding and maturation and are then transferred by ER-exit vesicles to the Golgi for additional processing and eventual secretion from the plasma membrane. Large protein complexes such as those formed by extracellular matrices and hormones require that monomer concentrations in the ER remain low to inhibit polymerization or aggregation. For such cargo, diffusion into the lumen of ER-exit vesicles alone (bulk flow) is insufficient, and more efficient trafficking is required, necessitating binding to ER-exit vesicle transmembrane cargo receptors. We show that amino-terminal tripeptides of several secreted proteins bind to transmembrane cargo receptor, surfeit locus protein 4 (Surf4), for increased ER-trafficking efficiency. Yeast’s homolog, ER-derived vesicles protein 29 (Erv29p), is functionally conserved, as it could rescue efficient ER trafficking in Surf4-null human cells. Proteome database analyses of candidate secreted proteins and direct experimentation with representative cargo proteins uncovered a range of tripeptide compositions that confer high, modest, or low efficiency of ER trafficking. Furthermore, many soluble chaperone proteins that function within the ER—as well as proteins too large to enter classic coat protein complex II (COPII) ER-exit vesicles, such as fibrillar collagens—start with tripeptides that cannot bind to Surf4/Erv29p. When cargo concentrations exceeded Surf4 levels, trafficking of problematical proteins was prioritized by use of their higher affinity ER-Exit by Soluble Cargo using Amino-terminal Peptide-Encoding motifs (ER-ESCAPE motifs).

## Introduction

Approximately one-half of proteins encoded within the human genome start with a leader sequence and/or encode transmembrane domains, suggesting they are translocated into the endoplasmic reticulum (ER) for trafficking to other organelles or into the extracellular environment [1]. Many soluble proteins translocated into the ER are destined to form polymers for a variety of evolutionary reasons, including assembling extracellular matrices. Because many extracellular environment properties of higher eukaryotes (neutral pH, oxidative environment, approximately 1 mM Ca^2+^, etc.) are similar to that in the lumen of the ER [2], premature assembly of matrix components is possible. Even proteins such as hormones form temporary close-packed arrays within storage-type secretory vesicles. While controlled oligomerization of some proteins in the ER is apparently desired [3], other ER-associated interactions could form damaging aggregates within the lumen. The simplest solution to this problem is to remove problematic cargo proteins from the ER before they can accumulate to concentrations high enough to enable inappropriate or premature associations. Diffusion of cargo proteins from the ER lumen into cargo vesicles destined to the Golgi apparatus (“bulk flow”) is insufficient to keep many proteins at concentrations low enough to prevent aggregation. Cargo receptors have long been proposed to bind specific cargo proteins in coat protein complex II (COPII) exit vesicles for more efficient ER trafficking. Such cargo receptors are generally transmembrane proteins with lumenal domain(s) to bind the cargo while their cytosolic domain(s) interact with cytosolic COPII-vesicle proteins [4]. Since thousands of cargo exist, it is unlikely that cells have a unique cargo receptor for each problematic soluble cargo protein. Hence, cargo receptors must exist that recognize generic signals in proteins with similar trafficking specificities.

One protein that has high tendency to aggregate in the ER lumen and therefore must utilize a cargo receptor is dentin sialophosphoprotein (DSPP). To our knowledge, all nonsyndromic cases of Dentinogenesis Imperfecta (DGI) and the less severe dentin dysplasia (DD) are the result of dominant mutations in the *DSPP* gene encoding for DSPP. We have shown that mutant DSPP proteins failed to traffic out of the ER [5]. Many of the disease mutations change one of the first three amino acids of the mature protein that is left after removal of the leader sequence. These three amino acids encode for isoleucine-proline-valine (IPV) [5]. These changes were either direct, single-base missense mutations (P17L, P17S, P17T, or V18D) or mutations causing exon-3 to be skipped, also resulting in an acidic, isoleucine-proline-aspartic acid (IPD) amino-terminus. Because these mutations result in the more severe disease, DGI, we proposed that the amino-terminal IPV tripeptide was DSPP’s motif that bound an ER cargo receptor. Failure to interact with this unknown cargo receptor would cause IPD-DSPP proteins, with its fully intact Ca^2+^-binding repeat domain, to accumulate to higher steady-state concentrations in the Ca^2+^-rich ER and result in the formation of Ca^2+^-associated aggregation. Experiments in support of this showed that the amount of wild-type DSPP (IPV) secreted by human embryonic kidney cell line 293A (HEK293A) was inversely correlated with increasing amounts of coexpressed mutant protein (IPD, isoleucine-serine-valine [ISV], or isoleucine-threonine-valine [ITV]-DSPP) [5].

In this current work, we identify that indeed the first three amino acids of many secreted proteins form a tripeptide motif that enhances exit from ER. We call this the ER-Exit by Soluble Cargo using Amino-terminal Peptide-Encoding motif (ER-ESCAPE motif). Furthermore, we defined the location, size, and biochemical properties of the proposed ER-ESCAPE motif. We identify surfeit locus protein 4 (Surf4) and its yeast homolog, ER-derived vesicles protein 29 (Erv29p), as the cargo receptor in human/yeast cells (respectively) that binds to the ER-ESCAPE motif, thereby enhancing ER trafficking of specific soluble cargo proteins. We explore implications that variations of the motif result in different Surf4-binding affinities and different priorities in ER exit for substrates that have differential exit requirements.

## Results

### Defining required properties of IPV-like motifs for ER trafficking of DSPP and AMELX

**Fig 1:**
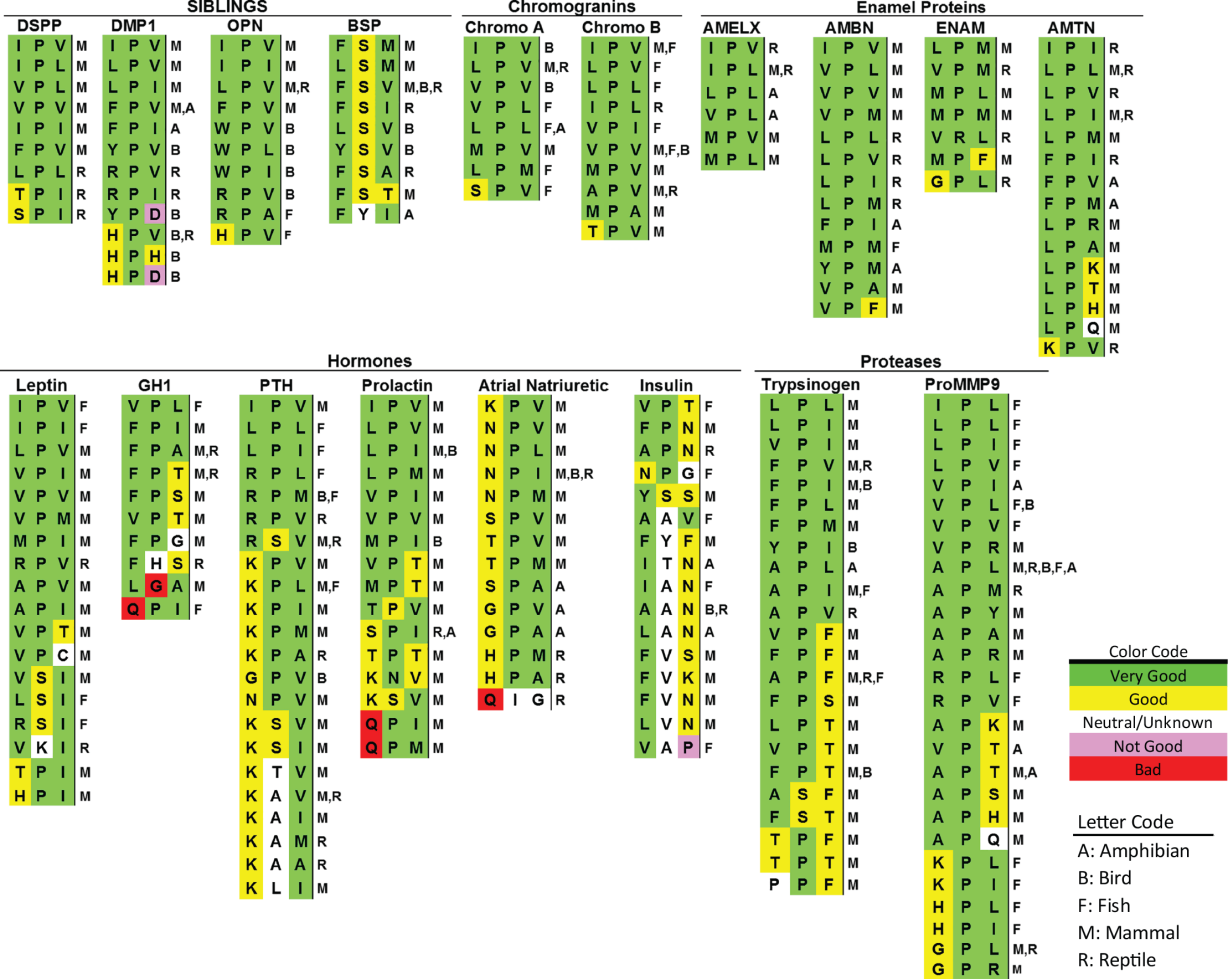
Examples of starting tripeptides for several classes of secreted vertebrate proteins with conserved, ER-ESCAPE motifs. All tripeptides found in NCBI Protein database searches (by gene name and BLASTP) are listed a single time for each protein, with vertebrate taxon notations in single-letter codes on the right. Signal peptide cleavage sites were predicted by SignalP 4.1 Server (http://www.cbs.dtu.dk/services/SignalP/) [6], Phobius (http://phobius.sbc.su.se/) [7], and/or experimental evidence noted on NCBI Proteins database. (See S1 Table for accession number, species name, and brief sequence of representative taxon for each tripeptide.) Color-coding based on relative contribution of each amino acid position to the strength of the ER-ESCAPE motif is as noted in Results and Discussion. AMBN, ameloblastin; AMELX, amelogenin, X-linked; AMTN, amelotin; BSP, bone sialoprotein; DMP1, dentin matrix acidic phosphoprotein 1; DSPP, dentin sialophosphoprotein; ENAM, enamelin; ER-ESCAPE motif, Endoplasmic Reticulum Exit by Soluble Cargo using Amino-terminal Peptide-Encoding motif; GH1, growth hormone 1; NCBI, National Center for Biotechnology Information; OPN, osteopontin; proMMP-9, pro-matrix metalloproteinase-9; PTH, parathyroid hormone.

Investigating the effectiveness of trafficking soluble cargo out of the ER for the 8,000 possible permutations of amino-terminal tripeptides was unrealistic. Therefore, the investigation was limited to select permutations of amino acid size, charge, and hydrophobicity/hydrophilicity. A survey of mammalian DSPP starting tripeptides (Fig 1, S1 Table) shows a consensus motif of hydrophobic-proline-hydrophobic (Φ-P-Φ) with both hydrophobic amino acids being limited to large ones; isoleucine, valine, leucine, and phenylalanine. For reptilian DSPP-like sequences, polar-but-uncharged amino acids—serine (python) or threonine (alligators and crocodiles)—were observed in the first position.

**Fig 2:**
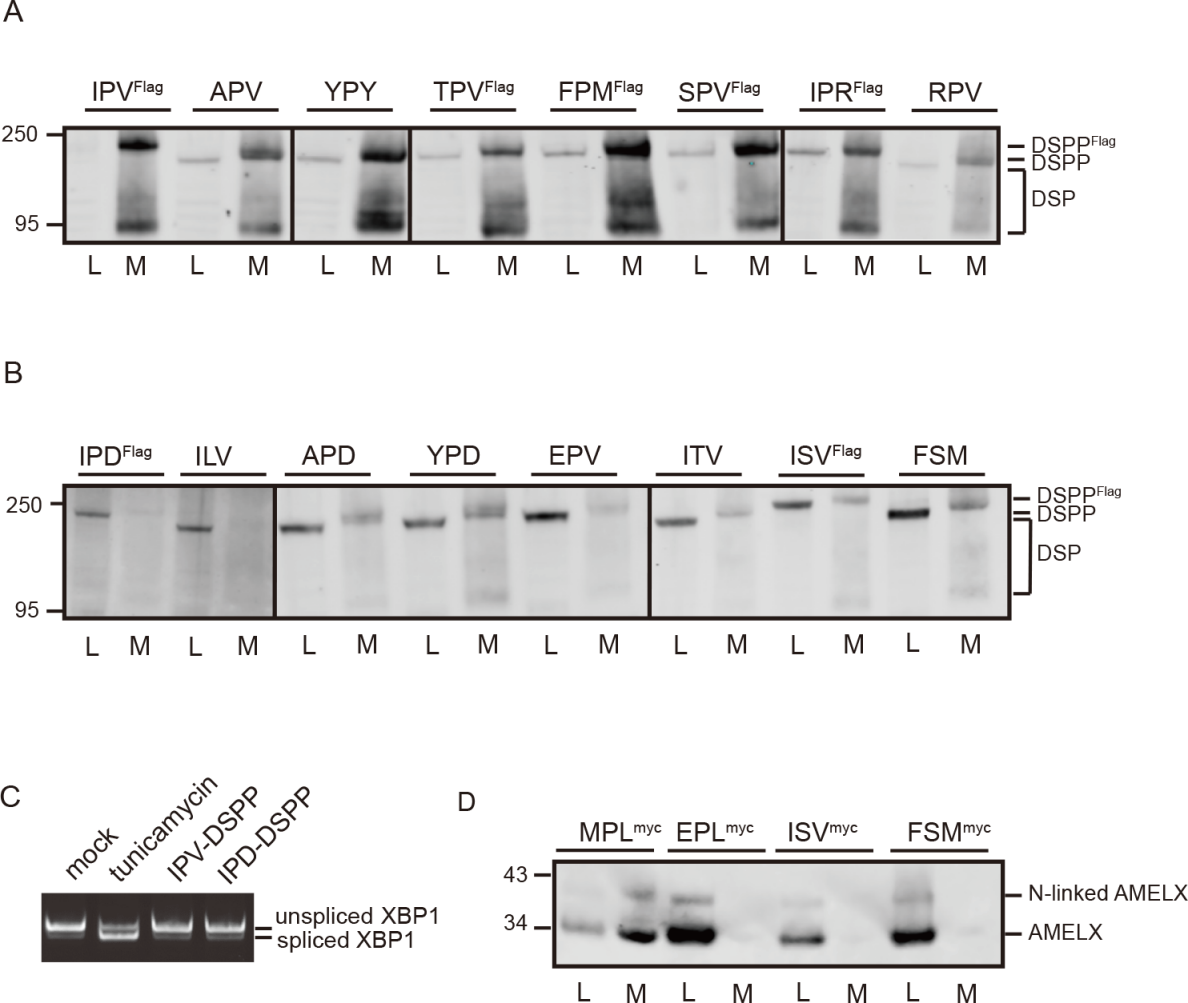
Composition of amino-terminal tripeptide is critical for ER trafficking of DSPP and AMELX in HEK293A cells. (A) Substitution of amino acids 1 and/or 3 of IPV with other hydrophobic (alanine, phenylalanine, methionine, or tyrosine), polar/uncharged (serine or threonine), or positively charged (arginine) amino acids in DSPP’s ER-ESCAPE motif continued to support efficient trafficking. Immunoblot shows protein levels of wild-type (IPV) and modified-tripeptide DSPP intact protein as well as cleavage product, DSP, in cell lysate (“L”) and conditioned media (“M”). Antibody to DSP domain was used for detection of intact DSPP as well as DSP-related cleavage products in the media. (B) Retention of DSPP in the cell shows trafficking of DSPP was disrupted by replacement of proline with leucine, serine, or threonine or substitution of either hydrophobic position with an acidic amino acid (D or E). Inclusion of 6xFlag-tag in noted constructs caused expected higher M_r_ of intact DSPP protein. (C) Mutations in DSPP’s IPV motif do not activate IRE1 pathway of the UPR (or ER stress). Note that 52 hr of expression of DSPP starting with wild-type (IPV) or mutant (IPD) did not cause the IRE1 pathway’s XBP-1 splice event associated with UPR. Addition of tunicamycin (known to activate UPR by inhibiting addition of N-linked oligosaccharides) for 5 hr resulted in abundant expression of the shorter, UPR-activating spliced XBP-1 message. (D) Wild-type (MPL) AMELX with carboxy-terminal Myc-tag was well trafficked out of the cell. AMELX with replacement tripeptide lacking number 2 position, proline (ISV or FSM), or replacement of starting hydrophobic with an acidic amino acid (EPL) all failed to traffic out of the cells. Faint band at higher M_r_ is due to small amount of AMELX that acquired a N-linked oligosaccharide. Anti-Myc antibody was used for detection. Cells were collected 18 hr (DSPP) or 24 hr (AMELX) posttransfection. Six μg of cell lysate protein or 20% of concentrated media was used for western blot analyses. Detection was with LI-COR Odyssey using IR-labeled second antibodies. Numbers on left are molecular weight standards in kDa. AMELX, amelogenin, X-linked; APD, alanine-proline-aspartic acid; APV, alanine-proline-valine; DSP, dentin sialoprotein; DSPP, dentin sialophosphoprotein; EPL, glutamic acid-proline-leucine; EPV, glutamic acid-proline-valine; ER, endoplasmic reticulum; ER-ESCAPE motif, ER-Exit by Soluble Cargo using Amino-terminal Peptide-Encoding motif; FPM, phenylalanine-proline-methionine; FSM, phenylalanine-serine-methionine; HEK293A, human embryonic kidney cell line 293A; ILV, isoleucine-leucine-valine; IPD, isoleucine-proline-aspartic acid; IPR, isoleucine-proline-arginine; IPV, isoleucine-proline-valine; IR, infrared; IRE1, inositol-requiring enzyme 1; ISV, isoleucine-serine-valine; ITV, isoleucine-threonine-valine; MPL, methionine-proline-leucine; RPV, arginine-proline-valine; SPV, serine-proline-valine; TPV, threonine-proline-valine; UPR, unfolded protein response; XBP-1, X-box-binding protein 1; YPD, tyrosine-proline-aspartic acid; YPY, tyrosine-proline-tyrosine.

Mouse DSPP expression constructs (with first 31 amino acids replaced by the human sequence as well as a 6xFLAG tag, when noted), starting with wild-type tripeptide (IPV) or noted tripeptides directly after the leader peptide, were used to test ER trafficking. HEK293A cells express a limited amount of bone morphogenetic protein 1 (BMP1) that cleaves DSPP into dentin sialoprotein (DSP) and dentin phosphoprotein (DPP) fragments during secretion [8]. Therefore, antibody against mouse DSP domain on western blots resulted in both intact (M_r_ approximately 200 kDa) and DSP fragments (broad M_r_ approximately 80 kDa) bands in culture media. When serine (serine-proline-valine [SPV]) or threonine (threonine-proline-valine [TPV]) were substituted for the starting isoleucine (IPV), DSPP was trafficked out of the ER and into the conditioned media as effectively as wild-type IPV-DSPP (Fig 2A).

Because it seemed unlikely that the proposed ER’s soluble cargo-trafficking receptor had evolved solely to traffic DSPP (a protein found only in creatures with complex, dentin-based tooth structures [9]), starting tripeptides of other acidic secreted proteins were queried next (Fig 1). Like DSPP, these very acidic proteins could be expected to form problematic, Ca^2+^-driven aggregates within the ER lumen if not kept to low concentrations by use of a high-affinity ER cargo receptor. The small hydrophobic amino acid, alanine, was found in the first position of chromogranin B (alanine-proline-valine [APV]) in some mammals and reptiles as well as the third position of some fish osteopontin (OPN; arginine-proline-alanine [RPA]) and reptile bone sialoprotein (BSP; phenylalanine-serine-alanine [FSA]). Alanine in the first position (APV) did enable efficient DSPP trafficking out of the ER (Fig 2A). Unpredicted from DSPP evolutionary data, the positively charged amino acid arginine was found starting dentin matrix acidic phosphoprotein 1 (DMP1) proteins of some reptiles (arginine-proline-valine [RPV], arginine-proline-leucine [RPL]) as well as OPN of specific fish (RPL, RPA, RPV, arginine-proline-glycine [RPG]) and one hummingbird (RPV).

Experimentally, DSPP was trafficked efficiently out of HEK293A cells when the positively charged arginine was in the first (RPV) or third (isoleucine-proline-arginine [IPR]) position (Fig 2A). As noted earlier [5], loss of proline in position 2 caused loss of trafficking (Fig 2B). Furthermore, acidic amino acids aspartate (D) or glutamate (E) in the first or third positions of the motif had similar negative effects (Fig 2B). DSPP lacks conserved hydrophobic domains found in stably folded proteins and therefore is like two other small integrin-binding ligand, N-linked glycoprotein (SIBLING) family members, BSP and OPN, which have been shown by NMR to be disordered (unstructured) proteins [10]. Indeed, Fig 2C shows that the accumulation of IPD-DSPP in the ER did not activate the IRE-1 unfolded protein response (UPR, ER-stress) pathway within 52 hr of transfection. In contrast, only 5 hr of treatment with tunicamycin (inhibits N-glycosylation needed by many proteins for correct folding) induced the alternate X-box-binding protein 1 (XBP-1) splice event of inositol-requiring enzyme 1 (IRE1)/UPR pathway. Of course, ER aggregates formed by other soluble cargo proteins could induce UPR pathways and contribute to negative evolutionary selection after loss/change of a beneficial amino-terminal tripeptide.

Our earlier work noted reports that several acidic proteins associated with mineralized matrices made by creatures as diverse as vertebrates, sea urchins, mollusks, and corals start with variations on the IPV motif, suggesting that trafficking acidic proteins via an ER cargo receptor was an ancient process [11]. Logically, however, any protein that is destined to form homopolymers (e.g., extracellular matrices and temporary aggregates in hormone/enzyme storage granules) would also require low ER concentrations to obviate aggregate formation. Furthermore, this hypothesis should include combinations of proteins destined to coassemble into complexes. The first line of defense against premature aggregation is by receptor-directed trafficking out of the ER. AMELX constitutes approximately 90% of the temporary extracellular matrix essential for enamel formation[12, 13]. The isoelectric point of AMELX is neutral and is unlikely to bind/share calcium ions like acidic proteins. However, upon secretion, AMELX self-associates into large structures [14, 15]. AMELX of mammals, reptiles, and amphibians (Fig 1) starts with a Φ-P-Φ, which, like DSPP, uses exclusively larger hydrophobic amino acids to flank the invariant proline.

Experimentally, we show that AMELX starting with the native tripeptide, methionine-proline-leucine (MPL), was efficiently trafficked out of HEK293A cells (Fig 2D). Replacement of proline with serine (ISV, phenylalanine-serine-methionine [FSM]) or starting the protein with an acidic amino acid, glutamic acid-proline-leucine (EPL), caused cellular retention of AMELX (Fig 2D). Use of the proposed ER cargo receptor, therefore, is likely not limited to acidic proteins that aggregate in millimolar Ca^2+^.

### The binding to cargo receptor is limited to the first three amino acids

Several protein hormones known to condense within secretory granules start with IPV-like tripeptides (Fig 1). We next investigated trafficking of growth hormone (GH) because of its ability to accumulate to higher levels than DSPP or AMELX in the ER of HEK293A cells before forming aggregates. Crystal structures of GH show that its amino-terminus is available for binding [16]. GH is also devoid of both N-linked oligosaccharides and glycophosphatidylinositol (GPI) modifications and therefore cannot utilize ER-Golgi intermediate compartment 53 protein (ERGIC-53)/lectin mannose-binding 1 (LMAN1) or p24 ER cargo receptors, respectively [17, 18]. GH is also sufficiently small (about 26 kDa) to be unlikely to interact with transport and Golgi organization 1 (TANGO1)/cTAGE5-associated large-cargo exit vesicles [19, 20].

**Fig 3:**
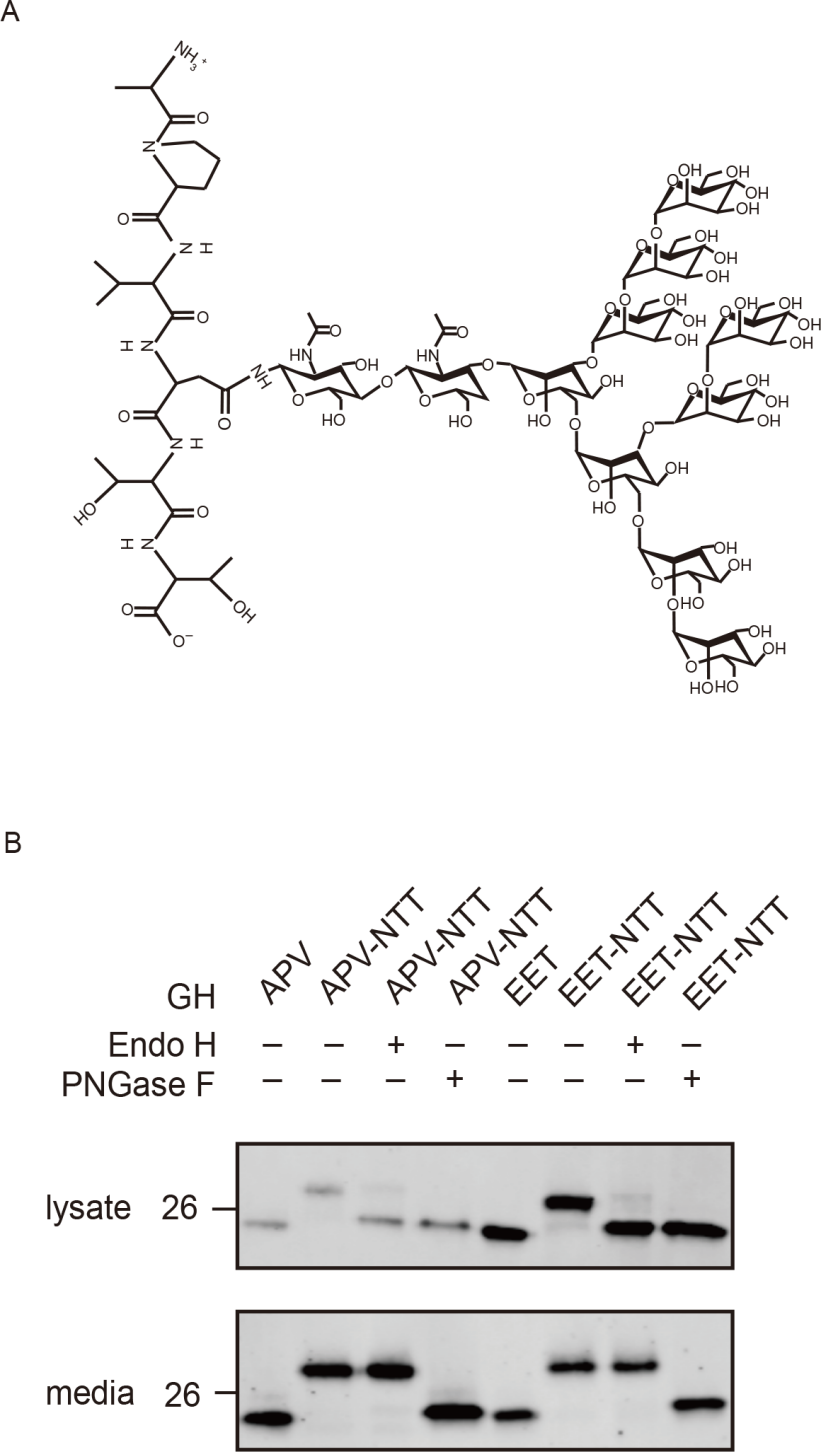
Addition of N-linked oligosaccharide at fourth position does not change efficiency of amino-terminal tripeptide-enhanced GH trafficking. (A) Chemical structure of N-linked glycosylation on APVNTT peptide (amino-terminal alanine at top). (B) Western blot: GH starting with high-efficiency motif, APV, obtained lower steady-state levels in HEK293A cells compared to same protein starting with predicted nonbinding motif, EET. Addition of N-linked oligosaccharide (NTT motif inserted at position 4) caused PNGaseF-susceptible increases in size (M_r_) but did not change relative trafficking efficiencies of either strong (APV-NTT) or nonbinding (EET-NTT) proteins. The oligosaccharides on cell layer–associated GH by both constructs were removed by Endo H digestion, showing no Golgi modifications. Cells and media were collected 16 hr posttransfection. Three μg of cell lysate or 6% of concentrated media was used for western blot analysis with goat anti-human GH. LI-COR IR-fluorescent second antibodies were used for detection on LI-COR’s Odyssey scanner. Numbers on left are molecular weight standards in kDa. APV, alanine-proline-valine; EET, glutamic acid–glutamic acid–threonine; GH, growth hormone; HEK293A, human embryonic kidney cell line 293A; IR, infrared; NTT, asparagine-threonine-threonine.

To query the fourth amino acid’s importance, an asparagine-threonine-threonine (NTT) N-linked oligosaccharide-attachment motif was inserted in position 4 of GH (APVNTT-GH). This oligosaccharide is several times the mass of IPV-like tripeptide itself (Fig 3A). The majority of APVNTT-GH was glycosylated by HEK293A cells, and its steady-state concentration in the cell remained as low as GH lacking the modification (APV-GH, Fig 3B). Because N-linked oligosaccharide addition to GH may have enabled ER trafficking by ERGIC-53/LMAN1, a control cargo protein (glutamic acid-glutamic acid-threonine [EET]-GH) was tested. It has two acidic amino acids and loss of position 2 proline, conditions that reduce/destroy ER trafficking of DSPP and AMELX. Consistent with an inability to interact with HEK293A’s IPV-motif cargo receptor, EET-GH established a much higher intracellular concentration than APV-GH (Fig 3B). Addition of an N-linked oligosaccharide (EETNTT-GH) did not decrease steady-state levels, suggesting that ERGIC-53/LMAN1 could not replace the IPV-motif ER-exit pathway. Endo H removes N-linked oligosaccharides from proteins found within the ER but has no effect on Golgi-modified proteins. All forms of the oligosaccharide can be removed by PNGase F. Susceptibility of both APVNTT-GH and EETNTT-GH in cell lysates (but not from media) to Endo H showed that most cell-associated GH remained within the ER (Fig 3B). These results present strong evidence that the IPV-like motif is limited to the first three amino-terminal amino acids. The abundance of secreted EET-GH suggests that HEK293A has a robust ER-Golgi “bulk flow” process.

### Identification of yeast ER-cargo receptor that interacts with proteins starting with IPV-like motifs

**Fig 4:**
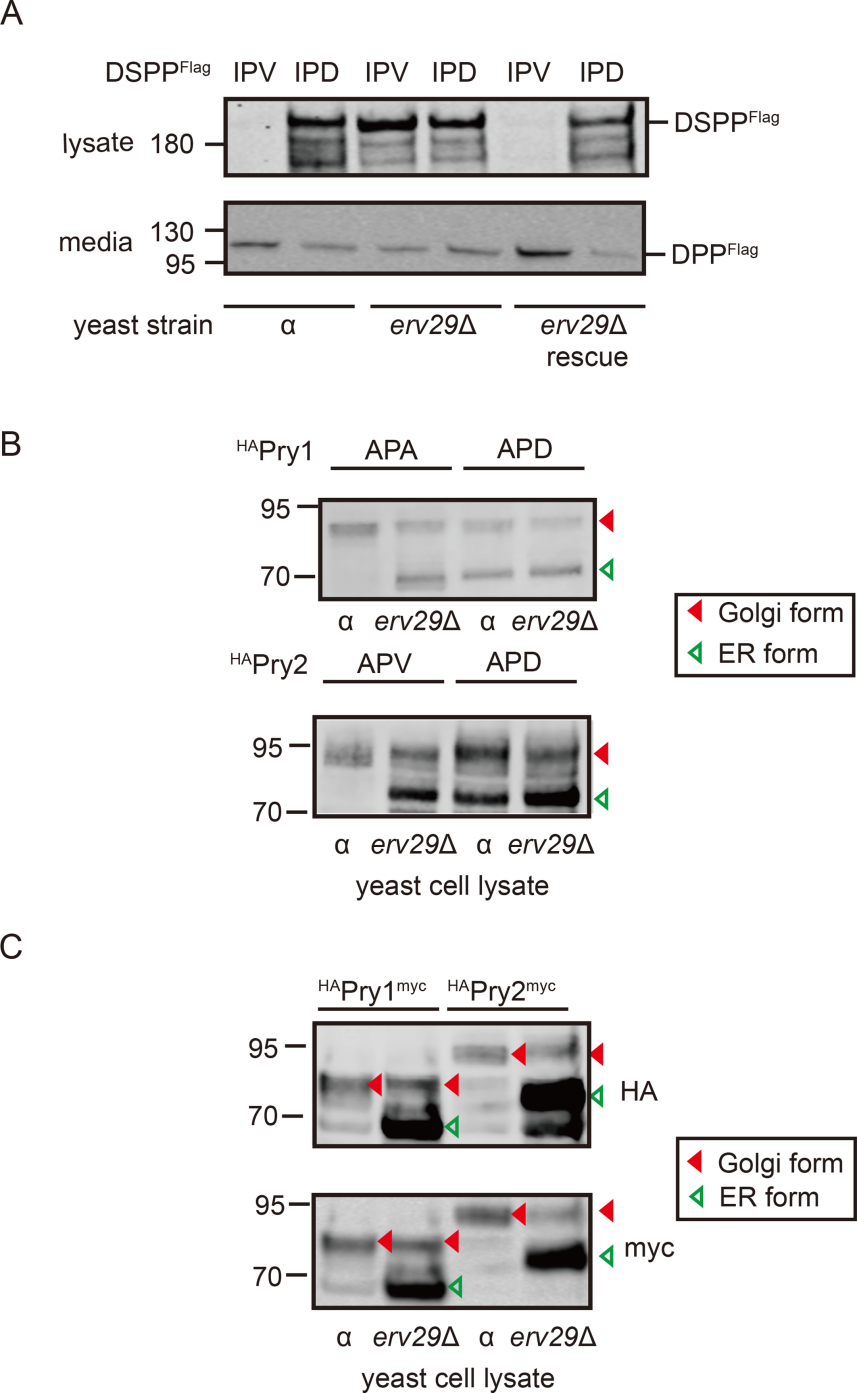
Erv29p is critical for trafficking of DSPP and PRY-family proteins in *S*. *cerevisiae*. (A) Immunoblot of wild-type DSPP^Flag^ (IPV) or DSPP^Flag^ starting with the mutant tripeptide motif (IPD) in α or *erv29*Δ cell lysates. Yeast α or *erv29*Δ cells were transformed with pYES expression plasmid encoding IPV- or IPD-DSPP^Flag^ under GAL1 promoter and induced for 5 hr. For rescue experiments, pAG425GPD plasmid constitutively expressing Erv29p was cotransformed with DSPP-encoding plasmids. Note that IPV-DSPP^Flag^ was well trafficked out of the ER/cells of wild-type α cells, while IPD-DSPP^Flag^ protein was retained. The *erv29*Δ cells could not efficiently traffic IPV-DSPP^Flag^ protein unless it was cotransformed with Erv29p-expressing plasmid (rescue). Media contained Kex2-digested, Flag-tagged carboxy-terminal fragment DPP. Four μg of cell lysate protein and 10% of the concentrated conditioned media were used for Flag-tag detection on western blots. (B) Yeast wild-type α cells or *erv29*Δ cells were transformed with pYES expression plasmids encoding Pry1p or Pry2p, each with 2xHA-tags near the amino-terminus. Five hr after induction, 30 μg of cell lysate protein was analyzed by western blots. For each construct, higher M_r_ bands (solid red arrowheads) were proteins containing Golgi-acquired posttranslational modifications, while ER-retained proteins lack these modifications and electrophorese several kDa faster (open green arrowheads). Note both APA/APV and APD showed increases in the smaller ER forms in erv29Δ cells, while only APD shows abundance in ER forms in wild-type (α) cells. (C) Pry1p and Pry2p modified to express both 2xHA-tag (near amino-terminus) and Myc (carboxy-terminus) showed faster electrophoresing proteins (ER) were not due to endogenous protease activity. LI-COR IR-fluorescent second antibodies were used for detection on LI-COR’s Odyssey scanner. Numbers on left are molecular weight standards in kDa. APA, alanine-proline-alanine; APD, alanine-proline-aspartic acid; APV, alanine-proline-valine; DPP, dentin phosphoprotein; DSPP, dentin sialophosphoprotein; ER, endoplasmic reticulum; Erv29p, ER-derived vesicles protein 29; HA, hemagglutinin; IPD, isoleucine-proline-aspartic acid; IPV, isoleucine-proline-valine; IR, infrared; Kex2, kexin 2; PRY, pathogen-related yeast.

*Saccharomyces cerevisiae* has long been an experimental model for basic eukaryotic cell functions, including ER-Golgi trafficking. Haploid wild-type alpha (α) cells made and secreted IPV-DSPP, while IPD-DSPP was predominantly retained within cells (α lanes, Fig 4A). The amino-terminus of the carboxy-terminal fragment observed in the media was found by microsequencing to start with asparagine-serine-proline [NSP], suggesting that cleavage was the result of the yeast’s endogenous Golgi propeptidase, kexin 2 (Kex2p), at the aspartic acid-lysine-arginine [DKR]-NSP motif near the BMP1 cleavage domain in higher eukaryotes [8]. Clones were selected from an α cell knockout (KO) library that were lacking one of 12 ER-associated, transmembrane proteins considered reasonable candidates for being ER cargo receptors (24 kDa endomembrane protein [Emp24p], 46 kDa endomembrane protein [Emp46p], 47 kDa endomembrane protein [Emp47p], erythrocyte protein 1 [Erp1p], erythrocyte protein 2 [Erp2p], ER-derived vesicles protein 14 [Erv14p], ER-derived vesicles protein 15 [Erv15p], ER-derived vesicles protein 25 [Erv25p], ER-derived vesicles protein 26 [Erv26p], Erv29p, ER-derived vesicles protein 46 [Erv46p], retention of ER proteins 1 [Rer1p]). Each KO strain was modified to express either IPV-DSPP or IPD-DSPP, and only the *erv29*Δ strain gave results predicted for loss of ER-to-Golgi receptor (S1 Fig). The clone lacking *ERV29* gene poorly trafficked both IPV-DSPP and mutant IPD-DSPP proteins (*erv29*Δ lanes, Fig 4A). Importantly, trafficking of IPV-DSPP but not IPD-DSPP was rescued when each was expressed in the *erv29*Δ cells cotransformed with second plasmid constitutively expressing Erv29p (rescue lanes, Fig 4A).

To verify that mammalian DSPP results were not just an expression artifact in yeast, two pathogen-related yeast (PRY) proteins starting with Φ-P-Φ motifs, Pry1p (APA) and Pry2p (APV), were expressed in wild-type and *erv29*Δ cells. Pry1p and Pry2p are both acidic (pI = 3.4), sterol-binding proteins said to reside in the cell for a time to bind toxic sterols before being secreted [21]. Both proteins were labeled near the amino-terminus with a 2xHA-tag. Pry1p in wild-type cells (α) was predominantly an M_r_ approximately 85 kDa band (Fig 4B). Pry1p expressed in *erv29*Δ cells resulted in some M_r_ approximately 85 kDa band but much more M_r_ approximately 70 kDa band. The smaller band is consistent with an ER-retained protein (i.e., before Golgi-related posttranslational modifications were added). Similarly, Pry2p protein in wild-type cells was predominantly the larger, Golgi-modified size (M_r_ about 95 kDa), while the smaller ER form (M_r_ approximately 75 kDa) increased in the cells lacking Erv29p cargo receptors (Fig 4B). Furthermore, changing the starting tripeptide of Pry1p and Pry2p to alanine-proline-aspartic acid (APD) resulted in increased ER-associated M_r_ bands of both wild-type and *erv29*Δ cells (Fig 4B). Pry1p and Pry2p proteins modified to express both hemagglutinin (HA)-tag (near amino-terminus) and Myc-tag (carboxy-terminal) were detected by both HA-antiserum (top panel) and Myc-antiserum (bottom panel), thereby showing that the faster electrophoretic bands (ER-retained proteins) were not produced by endogenous proteases (Fig 4C). Therefore, Erv29p that was reported to be a soluble cargo receptor for several yeast proteins including Pry1p [22] and the acidic (pI = 4.8) mating hormone, pro-α-factor [23], likely uses IPV-like motif to bind cargo.

### Depletion of *Erv29* homolog, *Surf4*, in human cell line negates enhanced ER-Golgi trafficking for specific soluble cargo

**Fig 5:**
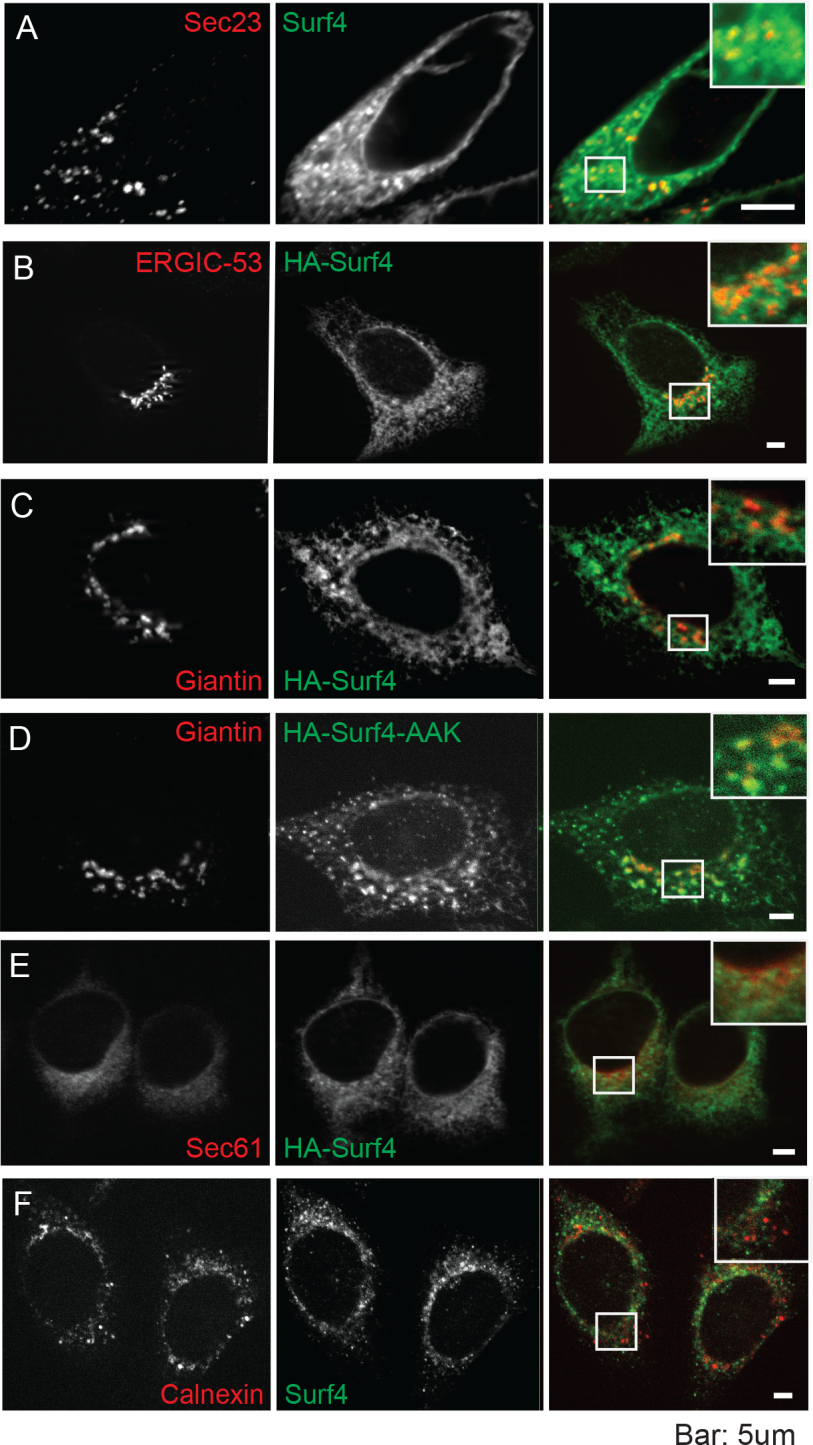
Immunofluorescence microscopy of HEK293A cells shows Surf4 accumulates in and around ERESs. (A) Fluorescent signal for endogenous Surf4 (green) was strongest at punctate structures positive for ERES marker Sec23 (red). Note additional Surf4 fluorescence in weblike structures surrounding ERES. (B) HA-Surf4 signal (green) was observed within the ERGIC (ERGIC-53, red). (C) HA-Surf4 (green) showed only low levels of colocalization with *cis*-Golgi marker, giantin (red). (D) Mutation of proposed COPI recycling motif by replacement of two of three near-carboxy-terminal lysines to alanines (HA-Surf4-AAK, green) increased colocalization with *cis*-Golgi marker, giantin (red). (E) Newly synthesized HA-Surf4 (green) was found at low levels in the rER (Sec61 marker, red). (F) Surf4 (green) did not colocalize with chaperone, calnexin (red), in the quality control domain. HEK293A cells were transfected with wild-type HA-tagged Surf4 plasmid (B, C, E), or *Surf4*^KO^ HEK293A cells were transfected with carboxyl-terminal di-lysine mutation, HA-*Surf4*-AAK (D), 18 hr prior to fixation. Bars = 5μm. The cells in each panel are shown 3 times, first with organelle marker, then Surf4 and final panel (with magnified insert) showing overlap. Alexa Fluor secondary antibodies were used for detection. Images were obtained using an LSM 780 (Carl Zeiss) confocal microscope (488 and 561 nm excitation lines; 500–560 and 600–660 nm capture) and Zeiss Axio Imager Z1 with Apotome 2 (single Z stack slice). Images were analyzed using Zeiss Zen software. AAK, alanine-alanine-lysine; COP, coat protein complex I; ERES, endoplasmic reticulum exit site; ERGIC, endoplasmic reticulum–Golgi intermediate compartment; HA, hemagglutinin; HEK293A, human embryonic kidney cell line 293A; rER, rough ER; Surf4, surfeit locus protein 4.

*Surf4*, the human homolog of *ERV29* [24], is considered a housekeeping gene in higher eukaryotes [25, 26]. Surf4 was expressed as a netlike structure including punctate colocalization with the ER exit site (ERES) marker, Sec23 (Fig 5A), and the ERGIC marker ERGIC-53, (Fig 5B). Even when overexpressed, recombinant Surf4 (HA-tagged) showed only low-to-modest residence in *cis*-Golgi (Fig 5C). However, as predicted [27], changing two of three lysines near the carboxy-terminus of Surf4 to alanines (proposed COPI vesicle-associated recycling motif) greatly increased its expression in *cis*-Golgi (Fig 5D, HA-Surf4-alanine-alanine-lysine [AAK]). There was little localization of HA-Surf4 in either the rough ER (rER) (Fig 5E) or ER quality control (QC) domain (Fig 5F), suggesting that newly synthesized Surf4 resides predominantly in the ERES and the microenvironment near actively forming COPII exit vesicles.

**Fig 6:**
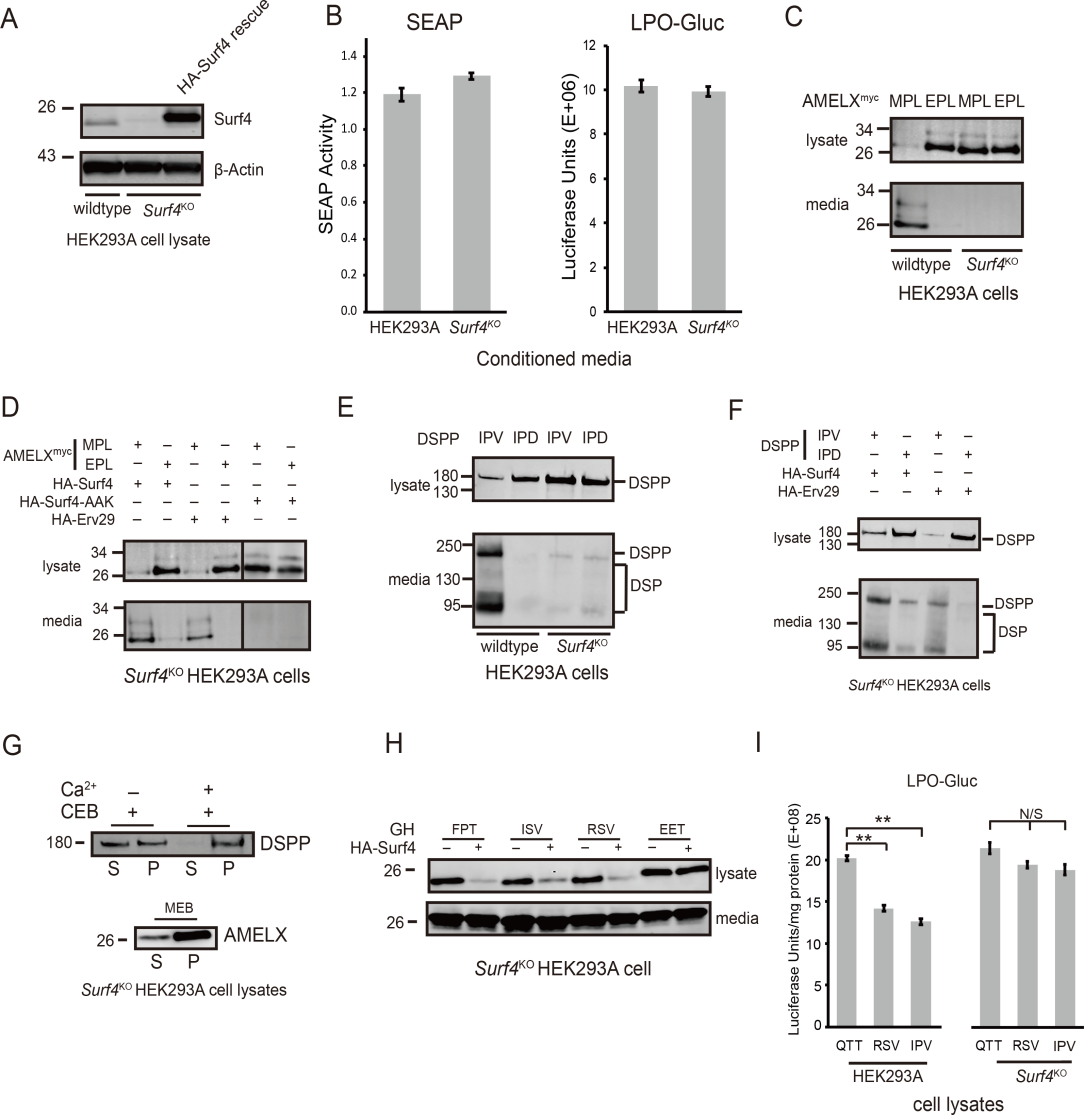
Surf4 is essential for keeping AMELX, DSPP, and GH at low concentrations within ER. (A) CRISPR/Cas9 technology was used to delete *SURF4* alleles from HEK293A cells (*Surf4*^KO^). Endogenous Surf4 was detected in HEK293A cell lysate (left lane) but not in *Surf4*^KO^ cell lysate (center lane). Right lane shows reexpression of Surf4 using plasmid-encoding HA-tagged Surf4 in *Surf4*^KO^. Introduction of HA-tag slightly increased the M_r_ of Surf4. Detection of Surf4 was with affinity-purified rabbit antibody to carboxy-terminal peptide. Lower panel: Detection of β-Actin serves as loading/protease controls. (B) Trafficking of secreted proteins lacking Surf4-binding motifs were unaffected by loss of Surf4. SEAP and LPO-Gluc were equally well secreted from normal and *Surf4*^KO^ cells. Conditioned media were harvested 22 hr posttransfection. SEAP secretion was assayed with 5μl of conditioned media using QUANTI-Blue kit. Luciferase activity was determined using 5 μl of conditioned media with BioLux Gaussia Luciferase Assay kit following Assay Protocol II. (Error bars are SEM with a transfection sample size of *n* = 5 [SEAP] and *n* = 6 [LPO-Gluc]) (C) AMELX^myc^ starting with MPL (Lane 1) well trafficked out of wild-type cells, but mutant EPL-AMELX (Lane 2) was not. Neither protein was efficiently trafficked out of *Surf4*^KO^ cells (Lanes 3 and 4). AMELX was detected using primary antibody to Myc-tag. (D) Trafficking of wild-type AMELX (MPL) in *Surf4*^KO^ cells was rescued by coexpression of either HA-Surf4 (Lane 1) or yeast’s Erv29p (Lane 3), but trafficking of EPL-AMELX was not rescued by either cargo receptor (Lanes 2 and 4). Coexpression of Surf4 lacking proposed motif for COPI recycling to ER (HA-Surf4-AAK) also could not rescue trafficking of MPL-AMELX (Lane 5). (E) Trafficking of IPV-DSPP in HEK293A cells (Lane 1) was lost in *Surf4*^KO^ cells (Lane 3). There was negligible trafficking of IPD-DSPP in either wild-type (Lane 2) or *Surf4*^KO^ cells (Lane 4). (F) Coexpression of HA-Surf4 (Lane 1) or HA-Erv29p (Lane 3) rescued IPV-DSPP trafficking in *Surf4*^KO^ cells but not for IPD-DSPP (Lanes 2 and 4). Primary antibody to mDSP domain was used to detect intact DSPP and its DSP fragment. (G) Evidence for aggregate formation by DSPP and AMELX (Myc-tagged) in *Surf4*^KO^ cells. Top panel: *Surf4*^KO^ cells expressing DSPP were briefly pelleted and then treated for 10 min with buffer containing digitonin (CEB) with (+) or without (-) 10 mM Ca^2+^ and pelleted at >100,000 x g. As observed on western blots, 10 mM Ca^2+^ stabilized a portion of DSPP in the pellet fraction. In the bottom panel, AMELX (Myc-tagged) formed stable aggregate in *Surf4*^KO^ cells with most remaining in >100,000 x g pellet after solubilizing cells with an MEB for 10 min. (H) Surf4-trafficked cargo with motifs other than Φ-P-Φ. Trafficking of GH lacking one hydrophobic amino acid (FPT), serine replacing proline at position 2 (ISV), or both lacking the proline, plus replacement of one hydrophobic with a positive-charged amino acid (RSV) were all rescued in *Surf4*^KO^ cells upon coexpression of HA-Surf4 protein. Trafficking of di-acidic EET-GH was not rescued by HA-Surf4. (I) LPO-Gluc, noted in Panel B as not using Surf4, acquired lower steady-state levels when wild-type motif QTT was replaced with strong ER-ESCAPE motifs (RSV or IPV). Same proteins expressed in *Surf4*^KO^ cells retained their high steady-state levels. Cells were harvested 22 hr posttransfection. The Luciferase activity was normalized to total protein (Luciferase units/mg protein). Error bars are SEM with sample size of *n* = 6 and *P* < 0.001 (**). For above experiments, cells were collected 18 hr (DSPP and GH) or 24 hr (AMELX) posttransfection. Ten μg of cell lysate protein and 20% of concentrated medium were used for western blots of DSPP and AMELX. GH analyses used 3 μg of cell lysate protein and 6% of concentrated media. LI-COR IR-fluorescent second antibodies were used for detection on LI-COR’s Odyssey scanner. Numbers on left are molecular weight standards in kDa. Φ-P-Φ, hydrophobic-proline-hydrophobic; AAK, alanine-alanine-lysine; AMELX, amelogenin, X-linked; AMELX^myc^, Myc-tagged human AMELX; Cas9, CRISPR-associated 9; CEB, Cytosol Extraction Buffer; COPI, coat protein complex I; CRISPR, clustered regularly interspaced short palindromic repeat; DSP, dentin sialoprotein; DSPP, dentin sialophosphoprotein; EET, glutamic acid–glutamic acid–threonine; EPL, glutamic acid-proline-leucine; ER, endoplasmic reticulum; ER-ESCAPE motif, ER-Exit by Soluble Cargo using Amino-terminal Peptide-Encoding motif; Erv29p, ER-derived vesicles protein 29; FPT, phenylalanine-proline-threonine; GH, growth hormone; HA, hemagglutinin; HEK293A, human embryonic kidney cell line 293; IPD, isoleucine-proline-aspartic acid; IPV, isoleucine-proline-valine; IR, infrared; ISV, isoleucine-serine-valine; LPO-Gluc human lactoperoxidase with carboxy-terminal luciferase; mDSP, mouse DSP; MEB, membrane extraction buffer; MPL, methionine-proline-leucine; N/S, not statistically significant; QTT, glutamine-threonine-threonine; RSV, arginine-serine-valine; SEAP, secreted alkaline phosphatase; Surf4, surfeit locus protein 4.

Using clustered regularly interspaced short palindromic repeat (CRISPR)/CRISPR-associated 9 (Cas9) technology, the alleles of *Surf4* were deleted in HEK293A cells (S2 Fig). Based on differences in sequence analyses, three independent colonies of *Surf4*^KO^ cells were obtained. Loss of Surf4 in the fruit fly is lethal [28], but cells of all three *Surf4*^KO^ clones were viable and had only slightly slower growth rates than their parent cells. A polyclonal antibody against the Surf4 carboxy-terminal cytosol domain detected the M_r_ approximately 26 kDa protein in wild-type HEK293A membrane extracts but not in *Surf4*^KO^ extracts (Fig 6A). *Surf4*^KO^ cells transfected with an expression plasmid encoding human HA-Surf4 rescued the protein’s expression.

Loss of Surf4 had no significant effect on the secretion of two model proteins lacking ER-ESCAPE motifs. Secretion of a commercially available, secreted form of alkaline phosphatase (SEAP, starting with native isoleucine-isoleucine-proline [IIP] tripeptide) or lactoperoxidase with a carboxy-terminal luciferase enzyme for analysis (LPO-Gluc; starting with glutamine-threonine-threonine [QTT]tripeptide) from both wild-type and *Surf4*^KO^ cells suggests no dramatic changes in critical components of the basic secretory pathway (Fig 6B).

As noted above, wild-type HEK293A cells could make and secrete wild-type AMELX while EPL- AMELX was retained inside the cell (Fig 6C). As would be expected if Surf4 is the Φ-P-Φ motif-binding ER cargo receptor, accumulation of wild-type MPL-AMELX equaled that of mutant EPL-AMELX protein construct in the *Surf4*^KO^ cells (Fig 6C). The efficient trafficking of native MPL-AMELX but not EPL-AMELX was rescued by coexpression of Surf4 in *Surf4*^KO^ cells (Fig 6D). Although only about 30% identical to human Surf4 protein (S3 Fig), coexpression of yeast homolog, Erv29p, also specifically rescued trafficking of AMELX starting with native MPL tripeptide in *Surf4*^KO^ cells (Fig 6D), thereby showing functional conservation in eukaryotic cells. Two lysine-to-alanine changes in the near-carboxy-terminus that caused Surf4 not to cycle back from Golgi to ER also did not rescue MPL-AMELX protein trafficking in *Surf4*^KO^ cells (Fig 6D, Surf4-AAK lanes). Similar results were found for DSPP constructs with *Surf4*^KO^ cells being unable to efficiently traffic either wild-type (IPV-DSPP) or mutant (IPD-DSPP; Fig 6E), while coexpression of the same proteins with either Surf4 or Erv29p rescued only DSPP starting with the intact IPV-motif (Fig 6F).

Fig 6G shows that wild-type IPV-DSPP accumulating in *Surf4*^KO^ cells is in the Ca^2+^-stabilized aggregate proposed earlier [5]. Permeabilizing intact cells for 10 min with Ca^2+^-free digitonin released about half of the DSPP into the >100,000 x g supernatant, while including 10 mM Ca^2+^ stabilized the DSPP aggregate such that it remained predominantly in the pelleted fraction. Wild-type MPL-AMELX aggregates in the *Surf4*^KO^ cells remained in the >100,000 x g pellet even in the presence of a membrane-solubilizing detergent (Fig 6G).

We next addressed whether Surf4 is a cargo receptor only for proteins starting with the Φ-P-Φ-like motif found in DSPP and AMELX. As the spectrum of human proteins starting with IPV-like tripeptides expanded from acidic, Ca^2+^-binding acidic proteins (e.g., SIBLINGs) to other matrix proteins known to self-aggregate in the extracellular environment (e.g., AMELX), as well as hormones and enzymes that aggregate temporarily in storage granules (e.g., GH), database searches were expanded to observe variation of IPV-related motifs occurring in proteins of other species. It was logical that some types of amino acids within tripeptides that did not keep DSPP or AMELX at sufficiently low concentrations within the ER to prevent aggregation may be successful for other proteins that form aggregates only at higher concentrations. For example, BSP is an acidic, calcium-binding SIBLING member with a well-conserved hydrophobic-serine-hydrophobic (Φ-S-Φ) in mammals, birds, and reptiles (Fig 1), even though a position number 2 serine for DSPP and AMELX resulted in loss of ER-to-Golgi trafficking. GH appeared to require a higher ER concentration to form aggregates in HEK293A cells and was successfully trafficked when started with tripeptide motifs other than Φ-P-Φ. ISV-GH and arginine-serine-valine (RSV)-GH both trafficked more efficiently in *Surf4*^KO^ cells when coexpressed with Surf4 (Fig 6H), illustrating the diversity of this receptor for different positive-binding motifs. Because secretion of LPO-Gluc was shown above to be unaffected by loss of Surf4 (Fig 6B), its starting tripeptide, QTT, was changed to two effective motifs (IPV or RSV) to see if intracellular steady-state levels could be decreased by addition of Surf4-interacting motifs. Fig 6I shows that starting lactoperoxidase (LPO) with either IPV or RSV significantly reduced their steady-state levels in HEK293A cells but not in *Surf4*^KO^ cells. Those results suggest that Surf4 is not only the cargo receptor for proteins starting with the Φ-P-Φ-like motif but also for proteins with differing compositions of the starting tripeptide motif. We propose use of the term ER-ESCAPE motif.

### Systematic mapping of ER-ESCAPE motif composition using GH as model soluble cargo

**Fig 7:**
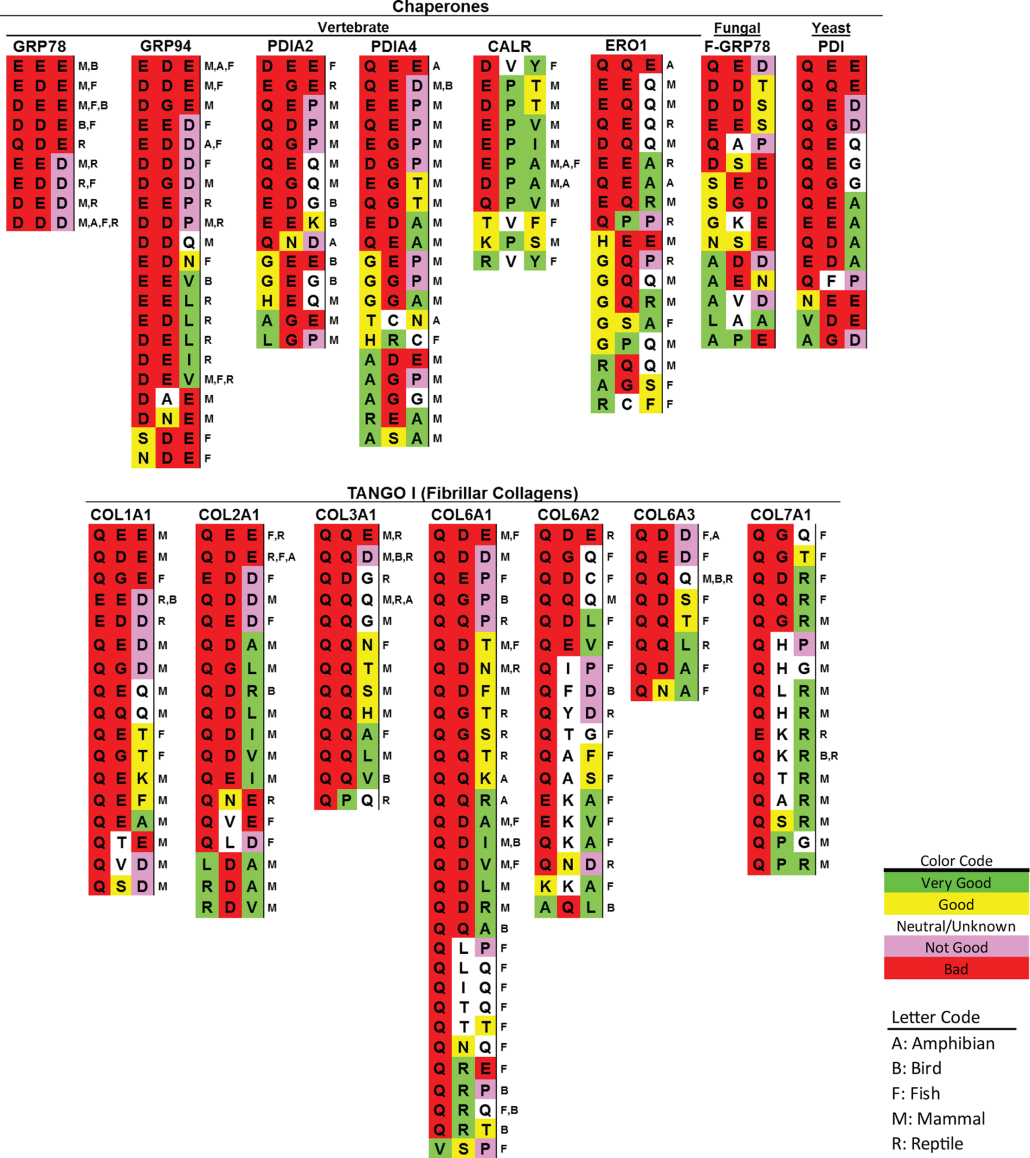
Examples of starting tripeptides in soluble proteins that are predicted not to interact with Surf4/Erv29p. After removal of leader sequences, amino-terminal tripeptides of TOP: vertebrate and fungal/yeast soluble ER-resident chaperone/modifying proteins and BOTTOM: vertebrate fibrillar collagens too large to fit in standard COPII exit vesicles. Each tripeptide found in NCBI Proteins database searches (by gene name or BLASTP) is listed with vertebrate taxon notations in single letter codes at right, indicating this tripeptide was found at least one time for this taxon (e.g., M = mammal). (See S2 Table for accession number, species name, and brief representative sequence.) Color-coding based on relative contribution of each amino acid position to strength of the ER-ESCAPE motif. CALR, calreticulin; COL1A1, collagen type 1 alpha 1; COL2A1, collagen type 2 alpha 1; COL3A1, collagen type 3 alpha 1; COL6A1, collagen type 6 alpha 1; COL1A2, collagen type 6 alpha 2; COL6A3, collagen type 6 alpha 3; COL7A1, collagen type 7 alpha 1; COPII, coat protein complex II; ER, endoplasmic reticulum; ER-ESCAPE motif, ER-Exit by Soluble Cargo using Amino-terminal Peptide-Encoding motif; ERO1, ER oxidoreductase 1; Erv29p, ER-derived vesicles protein 29; F-GRP78, fungal glucose-regulated protein 78; GRP78, glucose-regulated protein 78; GRP94, glucose-regulated protein 94; NCBI, National Center for Biotechnology Information; PDI, protein disulfide isomerase; PDIA2, PDI family A member 2; PDIA4, PDI family A member 4; Surf4, surfeit locus protein 4; TANGO 1, transport and Golgi organization 1.

Soluble chaperone proteins must remain in the ER to perform their functions, and any with exposed amino termini should have negligible binding affinities for Surf4/Erv29p. Our results from DSPP and AMELX experiments suggest that lack of proline in positions 2 and/or acidic amino acids in one or more of positions would decrease or even negate binding of proteins to Surf4/Erv29p. Chaperones glucose-related protein 78 (GRP78, or binding immunoglobulin protein [BiP]), glucose-related protein 94 (GRP94 [HSP90B1]), protein disulfide isomerase family A member 2 (PDIA2), protein disulfide isomerase family A member 4 (PDIA4), and calreticulin (CALR) across the eukaryotic evolutionary spectrum have at least one acidic amino acid within their starting tripeptide, and position 2 prolines are rare (Fig 7 and S2 Table). Similarly, vertebrate fibrillar collagens interact with TANGO1/cTAGE5-associated cargo receptor complexes to specifically direct them into alternative exit vesicles large enough to encapsulate them [19, 20]. Classic 60–70 nm COPII exit vesicles are too small for these large proteins. Therefore, any solvent-available starting tripeptides of large collagens should not interact with Surf4. Starting tripeptides of several large, fibrillar collagens of vertebrates (Fig 7, S2 Table) show at least one acidic amino acid or, curiously, one or two glutamines. The abundance of glutamines in position 1 and/or 2 (e.g., Type III collagen’s glutamine-glutamine-glutamine [QQQ], Fig 7) suggests that glutamines help establish motifs as Surf4 nonbinders.

**Fig 8:**
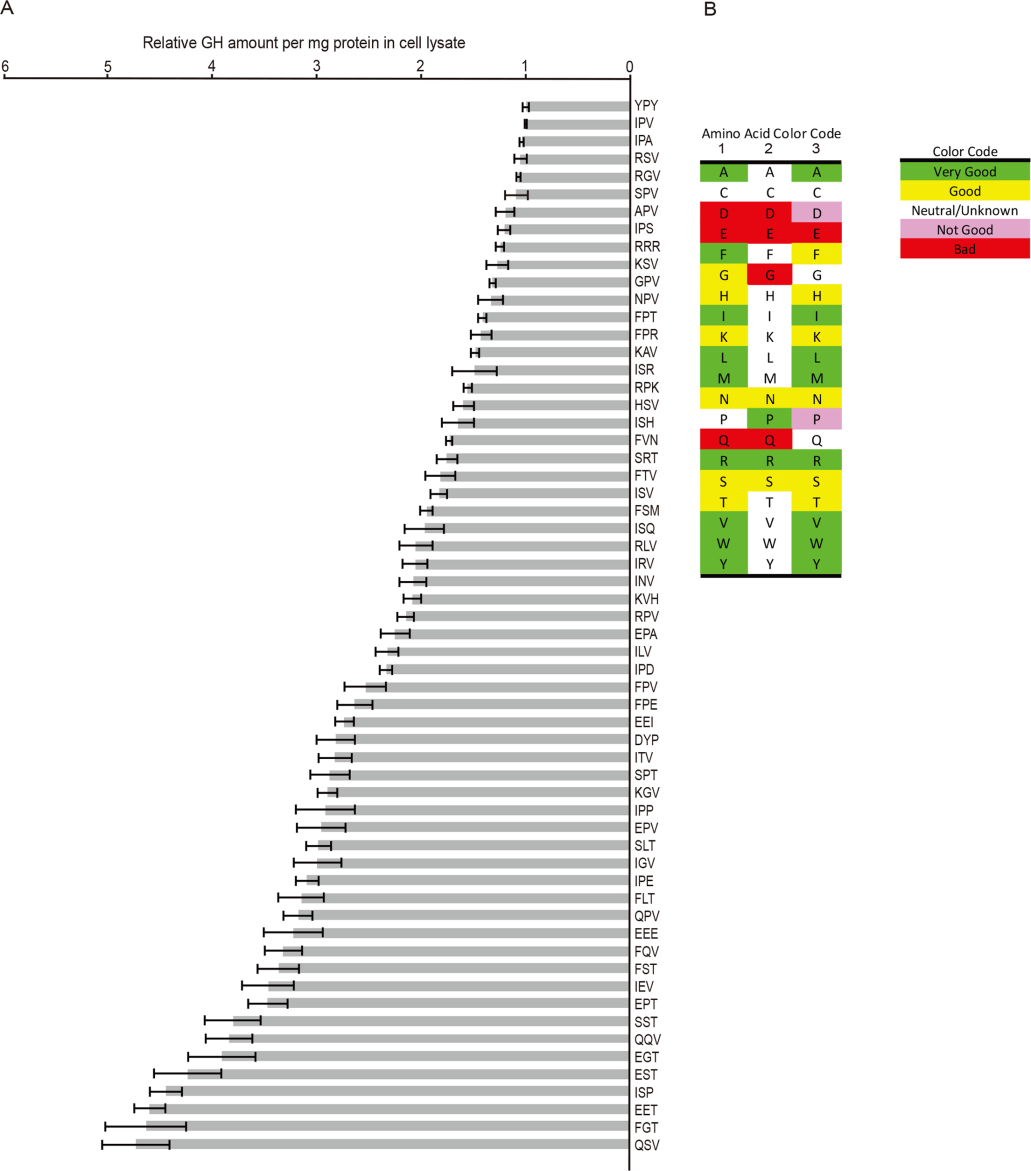
Relative affinities of ER-ESCAPE motifs for cargo receptor by analyses of GH steady-state levels. (A) Pre-confluent cells were separately transfected with expression plasmids encoding human GH with 60 different ER-ESCAPE motifs as noted. Eighteen hr posttransfection, cells were washed, extracted with lysis buffer, and analyzed by GH ELISA. GH values (ng GH/mg protein) were normalized within each experiment to the amount of IPV-GH. Each histogram bar represents mean ± SEM. of at least three independent transfections for each construct. (Means of triplicate GH ELISA analyses were used for each extract). Larger error bars among poorest ER-ESCAPE motifs may reflect a variable amount of aggregate formation by larger amounts of accumulated GH. Note that Φ-P-Φ ER-ESCAPE motifs are among the most efficient at trafficking GH, while those including acidic amino acids or glutamines are much less effective. Substitution by positively charged amino acids (generally, R better than K) retained effective trafficking, while loss of proline in position 2 was otherwise detrimental. (B) Prediction of value that each amino acid in positions 1, 2, and 3 adds to the quality of the ER-ESCAPE motif binding to Surf4 based on combination of ELISA and database search results. Note that some combinations may give results not predicted by simply summing a tripeptide’s three individual amino acids contributions indicated by this panel. Φ-P-Φ, hydrophobic-proline-hydrophobic; APV, alanine-proline-valine; DYP, aspartic acid-tyrosine-proline; EEE, glutamic acid–glutamic acid–glutamic acid; EEI, glutamic acid–glutamic acid–isoleucine; EET, glutamic acid–glutamic acid–threonine; EGT, glutamic acid-glycine-threonine; EPA, glutamic acid-proline-alanine; EPT, glutamic acid-proline-threonine; EPV, glutamic acid-proline-valine; ER-ESCAPE motif, ER-Exit by Soluble Cargo using Amino-terminal Peptide-Encoding motif; EST, glutamic acid-serine-threonine; FGT, phenylalanine-glycine-threonine; FLT, phenylalanine-leucine-threonine; FPE, phenylalanine-proline-glutamic acid; FPR, phenylalanine-proline-arginine; FPT, phenylalanine-proline-threonine; FPV, phenylalanine-proline-valine; FQV, phenylalanine-glutamine-valine; FSM, phenylalanine-serine-methionine; FST, phenylalanine-threonine-valine; FTV, phenylalanine-threonine-valine; FVN, phenylalanine-valine-asparagine; GH, growth hormone; GPV, glycine-proline-valine; HSV, histidine-serine-valine; IEV, isoleucine-glycine-valine; IGV, isoleucine-glycine-valine; ILV, isoleucine-leucine-valine; INV, isoleucine-asparagine-valine; IPA, isoleucine-proline-alanine; IPD, isoleucine-proline-aspartic acid; IPE, isoleucine-proline-glutamic acid; IPP, isoleucine-proline-proline; IPS, isoleucine-proline-serine; IPV, isoleucine-proline-valine; IRV, isoleucine-arginine-valine; ISH, isoleucine-serine-histidine; ISP, isoleucine-serine-proline; ISQ, isoleucine-serine-glutamine; ISR, isoleucine-serine-arginine; ISV, isoleucine-serine-valine; ITV, isoleucine-threonine-valine; KAV, lysine-alanine-valine; KGV, lysine-glycine-valine; KSV, lysine-serine-valine; KVH, lysine-valine-histidine; NPV, asparagine-proline-valine; QPV, glutamine-proline-valine; QQV, glutamine-glutamine-valine; QSV, glutamine-serine-valine; RGV, arginine-glycine-valine; RLV, arginine-leucine-valine; RPK, arginine-proline-lysine; RPV, arginine-proline-valine; RRR, arginine-arginine-arginine; RSV, arginine-serine-valine; SLT, serine-leucine-threonine; SPT, serine-proline-threonine; SPV, serine-proline-valine; SRT, serine-arginine-threonine; SST, serine-serine-threonine; Surf4, surfeit locus protein 4; YPY, tyrosine-proline-tyrosine.

Sixty starting tripeptides for GH were expressed in HEK293A and cell extracts analyzed by GH ELISA to determine steady-state levels. (Technical University of Denmark’s SignalP 4.1 predicted retained amino-terminal tripeptides.) Because not all trial permutations could be tested in a single experiment, candidate tripeptides’ results were normalized with respect to the representative well-trafficked ER-ESCAPE-motif IPV-GH included in each experiment (Fig 8A). This data confirmed earlier DSPP and AMELX results that a variety of Φ-P-Φ motifs established the lowest steady-state GH levels, as did positively charged arginines. When started with conserved BSP motif, Φ-S-Φ, FSM-GH had predicted modest steady-state level. This suggests that FSM, while not as good as a motif with a proline flanked by two hydrophobic amino acids, is successfully used by this Ca^2+^-binding, acidic protein to keep it from reaching its own problematic ER concentrations. While no harm would likely come to BSP acquiring a Φ-P-Φ motif, there appears to be evolutionary pressure to retain its modest-affinity motif, perhaps to keep from competing with more problematical cargo. Because GH can accumulate to higher levels in HEK293A before forming aggregates, this cargo model also highlighted that a single acidic amino acid within the tripeptide (e.g., IPD-GH, glutamic acid-proline-alanine [EPA]-GH) placed soluble cargo’s steady-state levels in mid to high ranges of ER-accumulation (i.e., low but positive affinity), whereas two or more acidic amino acids always resulted in highest steady-state levels (i.e., probably nonbinding).

The GH ELISA assay was also used to test the hypothesis that the large, uncharged/polar amino acid glutamine, often found in positions 1 and/or 2 of chaperones and other proteins reasonably expected to not bind Surf4/Erv29p (Fig 7), would accumulate to higher levels in cells. Indeed, GH starting with glutamine-proline-valine (QPV), glutamine-serine-valine (QSV), phenylalanine-glutamine-valine (FQV), or glutamine-glutamine-valine (QQV) were found at steady-state levels sufficiently high in cells to suggest weak or no binding to cargo receptor (Fig 8A). Chemically similar asparagine (R-group chain only one CH_2_ shorter than glutamine) did not substitute for glutamine in the database tripeptide sequences of chaperones or fibrillar collagens. Correspondingly, GH with asparagine in positions 1 (asparagine-proline-valine [NPV]-GH) or 2 (isoleucine-asparagine-valine [INV]-GH) resulted in low or modest GH steady-state levels, respectively, indicating stronger binding constants for Surf4 than similar tripeptides including glutamine.

As results of 60 tripeptide permutations were obtained, some guiding principles seemed to be reasonable: acidic amino acids and glutamine are bad for strong binding to Surf4; proline is good in position 2 but not 3; hydrophobic amino acids are good in positions 1 and 3 but not position 2, etc. (Fig 8B). However, levels of some tripeptide-GH proteins that such “rules” would seem to predict to be excellent binders did not always work as expected. For example, phenylalanine-proline-valine (FPV) is a Φ-P-Φ motif, but the FPV-GH construct had higher steady levels (modest affinity) than predicted (Fig 8A). Perhaps the binding pocket of Surf4 finds the combination of two of the largest hydrophobic amino acids flanking the proline to be slightly destabilizing. In another example, yeast aspartate protease (PrA) is a modestly acidic (pI 4.7) protein described as being dependent on Erv29p for ER-Golgi trafficking [24]. PrA starts with lysine-valine-histidine (KVH) tripeptide, a motif that, lacking a proline or serine in the number 2 position, did not fit the prediction model of a successful ER-ESCAPE motif. However, KVH-GH did have a positive, if modest, ability to enhance trafficking of GH out of the ER (Fig 8A). Therefore, the summary of our interpretation of the relative good/neutral/bad contribution of each of the 20 possible amino acids in positions 1, 2, and 3 in the amino-terminal tripeptide (Fig 8B) is generally useful for predicting strong or nonbinding motifs but is somewhat subjective for more modestly binding tripeptides.

**Fig 9:**
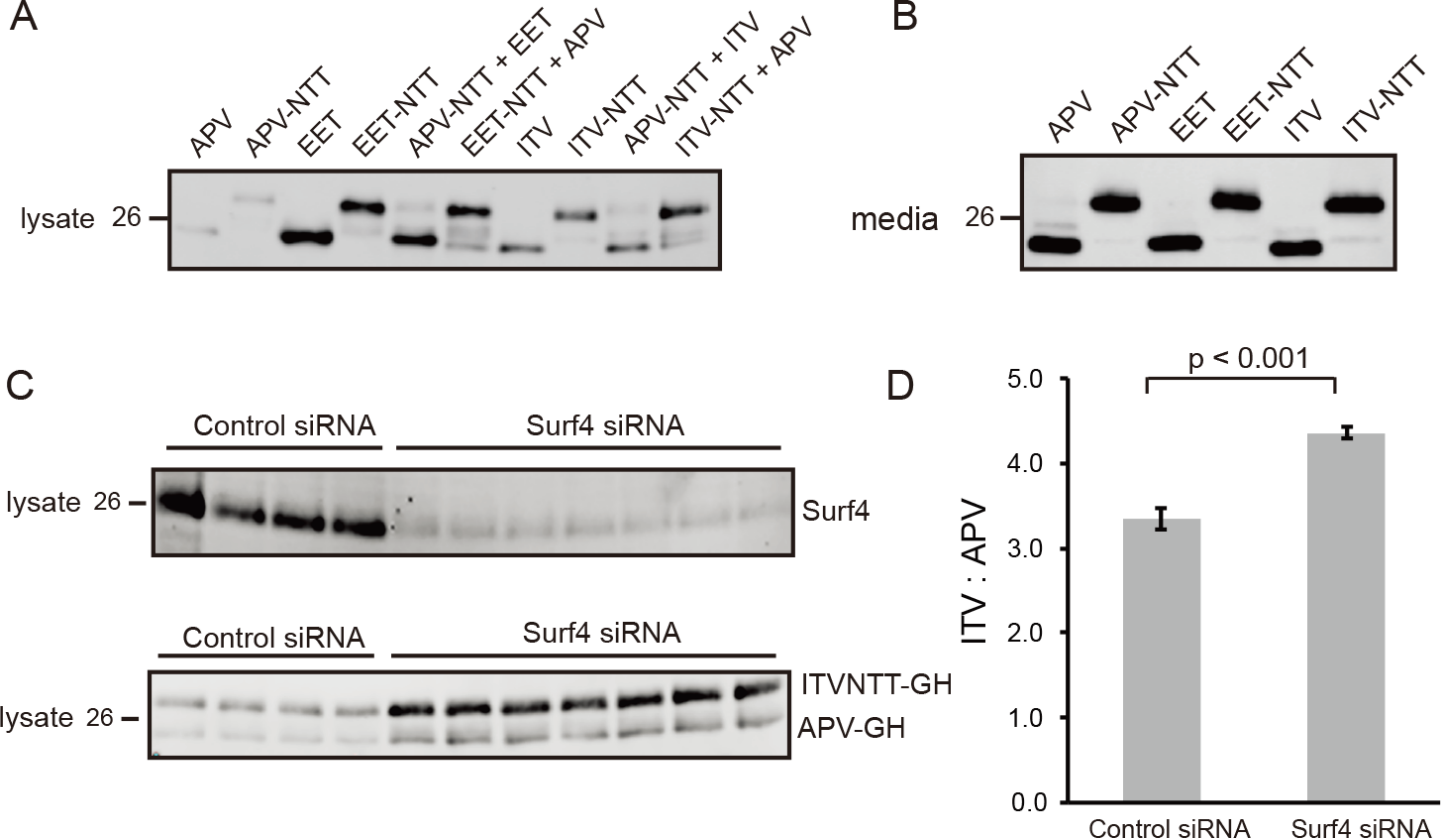
When not in functional excess, Surf4 prioritizes ER exit of cargo with stronger ER-ESCAPE motif. (A) Western blot analyses of cell extracts show steady-state levels of GH starting with strong ER-ESCAPE motif: APV < modest: ITV << nonbinding: EET. Addition of N-linked oligosaccharide (NTT) to fourth amino acid increased the mass/M_r_ but did not affect relative cellular steady-state levels for any of these proteins, (APV = APV-NTT) < (ITV = ITV-NTT) << (EET = EET-NTT). Coexpression of APV-GH and ITV-GH, irrespective of which construct was glycosylated, did not affect their respective steady-state levels. (B) GH detected in conditioned media (18 hr posttransfection) showed both the remaining complement of APV-GH was trafficked out of cells and the robust nature of HEK293A’s bulk flow process of nonbinding cargo (EET-GH). (C) Partial knockdown of Surf4 with siRNA depleted Surf4 protein levels sufficiently to show GH with strong binding ER-ESCAPE motif (APV-GH) outcompeting modestly binding GH (ITV-GH). HEK293A cells were pretreated for 36 hr with Surf4 siRNA before second transfection with noted GH constructs. Eighteen hr later, washed cells were processed for GH and Surf4 western blots. (D) Quantification of GH western blots shows modestly binding ITV-GH was less effectively trafficked than APV-GH when Surf4 levels were substantially depleted by siRNA. (Error bars are +/− SEM with sample size of *n* = 4 for inactive siRNA and *n* = 7 for Surf4 siRNA, *P* < 0.001.) Three μg cell lysate protein and 6% concentrated media were used for western blot analysis with goat anti-human GH. Six μg cell lysate protein was used for western blot analysis with rabbit anti-Surf4 carboxy-terminal peptide. LI-COR IR-fluorescent second antibodies were used for detection on LI-COR’s Odyssey scanner. Numbers on left are molecular weight standards in kDa. APV, alanine-proline-valine; EET, glutamic acid–glutamic acid–threonine; ER, endoplasmic reticulum; ER-ESCAPE motif, ER-Exit by Soluble Cargo using Amino-terminal Peptide-Encoding motif; GH, growth hormone; HEK293A, human embryonic kidney cell line 293; IR, infrared; ITV, isoleucine-threonine-valine; NTT, asparagine-threonine-threonine; siRNA, small interfering RNA; Surf4, surfeit locus protein 4.

When Surf4/Erv29p is in excess, each soluble cargo protein should come to an independent ER steady-state concentration based on its amino-terminal tripeptide’s receptor affinity. To test this hypothesis, GH starting with a well-trafficked motif, APV, was first separately expressed then coexpressed with either nontrafficking EET-GH or a modestly trafficking ITV-GH. To distinguish the two coexpressed GH proteins on western blots, one protein in each experiment was encoded with an N-linked oligosaccharide motif (APV-NTT, EET-NTT, ITV-NTT), causing it to electrophorese slower on western blots. To control for possible interactions between these carbohydrates and the lectin aspects of ERGIC-53/LMAN1 cargo receptor, each coexpression pairing was conducted twice, with expression of NTT motif alternating between the two proteins. The same amount of plasmid for each protein was used for transfection. Transfections involving a single construct included inert plasmid to make amounts of DNA equal. As expected from earlier GH ELISA results, western blots showed that a single expression of the strong ER-ESCAPE motif, APV-GH, established lowest steady-state concentration in cells, followed by modest levels of ITV-GH, while highest levels were observed with EET-GH whether these proteins contained an N-linked oligosaccharide or not (Fig 9A). Because GH steady-state levels +/− NTT were very similar when directly comparing results of each starting tripeptide (e.g., EET versus EET-NTT), GH trafficking by ERGIC-53/LMAN1 in HEK293A appears to be insignificant for GH. As noted earlier for SEAP and LPO-Gluc proteins (Fig 6B), bulk flow in HEK293A cells was robust, as significant secretion of GH into the media of all constructs including EET-GH was observed (Fig 9B). In contrast, bulk flow processes were unable to traffic similar amounts of DSPP or AMELX out of HEK293A cells because they have much a higher propensity to interact and were entrapped within aggregates shortly after expression commenced. Thus, monomeric DSPP and AMELX proteins apparently rarely escape being added to aggregates to diffuse to ERES domain for bulk flow ER trafficking. Cotransfection of expression plasmids differing only in starting tripeptides (plus NTT motif for one of each pair, as noted) was performed in HEK293A cells. The low steady-state levels of APV and APV-NTT GH proteins within the cell lysates remain unchanged when coexpressed with nonbinding EET-GH or the modestly binding ITV-GH proteins (Fig 9A). Because levels of ITV-GH when coexpressed with APV-GH protein also remained unchanged, Surf4 is in functional excess in HEK293A cells. The combination of robust bulk flow and expression of functional excess Surf4 even during overexpression of cargo by transfection may explain why HEK293A cells have long been used for recombinant protein production.

When HEK293A cells were treated for a total of 54 hr with Surf4-directed small interfering RNA (siRNA), Surf4 levels were significantly depleted compared to transfection with control siRNA (top panel, Fig 9C). Eighteen hr before harvesting, cells were transfected a second time with equal amounts of expression plasmids for APV-GH and the more modestly binding ITVNTT-GH. Under these conditions, levels of Surf4 were sufficiently low to become limiting and cause higher steady-state levels of both APV-GH and ITVNTT-GH (bottom panel, Fig 9C). However, quantification of new steady-state levels of GH in Surf4 knockdown cells resulted in the ITV:APV ratio being significantly increased (*P* < 0.001), showing that APV-GH was preferentially trafficked when Surf4 was not in excess (Fig 9D).

### Binding of GH with strong, modest, and poor enhanced-trafficking ER-ESCAPE motifs to Surf4 microsomes

**Fig 10:**
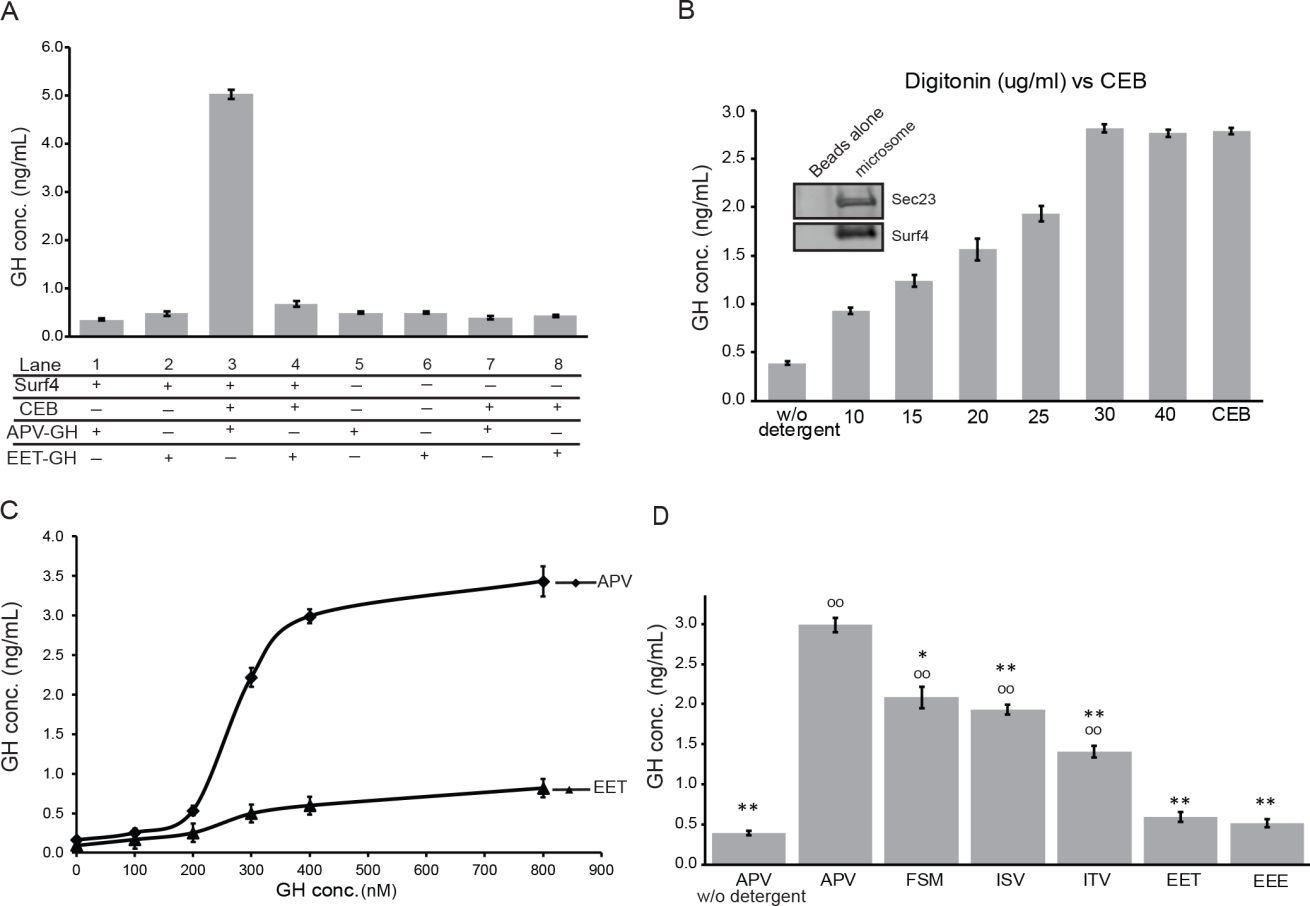
APV-GH interacts with digitonin-permeabilized Surf4 microsomes with half-maximal binding of 200–300 nM. (A) Microsomes made from *Surf4*^*KO*^ cells with (Lanes 1–4) or without (Lanes 5–8) expression of Surf4 expression by transfection for 24 hr. Half of the microsome aliquots (Lanes 3, 4, 7, and 8) were permeabilized with CEB (digitonin) for 30 min. As indicted, aliquots were incubated for 1 hr with 400 nM APV-GH or 400 nM EET-GH and briefly washed. Only combination of Surf4-expressing microsomes + digitonin + APV-GH resulted in significant increases (>5-fold) in detection by GH ELISA associated with the final >100,000 x g microsome pellet. (B) Preparation of microsomes from HA-Surf4-transfected HEK293A cells were incubated with magnetic beads precoated with antibodies to the cytosolic, carboxy-terminal domain of Surf4. Equal aliquots of Surf4 microsome beads were titrated with indicated concentration of digitonin (or CEB) for 30 min before incubation for 1 hr with 400 nM APV-GH, brief wash, and processing for GH ELISA analyses. Dose-response results show that CEB and ≥ 30 μg/ml digitonin were effective at permeabilizing microsomes for binding of APV-GH. Insert: Western blot shows that bead-associated microsomes contained both HA-Surf4 and the ERES marker, Sec23. (C) Equal aliquots of CEB-treated, Surf4 microsome/beads were incubated with increasing concentrations of APV-GH or EET-GH. APV-GH showed saturable binding characteristics with half-maximal binding at around 200–300 nM. EET-GH showed background levels of binding. (D) Equal aliquots of Surf4 microsome/beads were permeabilized with CEB (except first lane), incubated for 1 hr with 400 nM GH starting with indicated tripeptides, briefly washed, and analyzed by GH ELISA. Highest level of binding was with strong ER-ESCAPE motif APV-GH, followed by three modest binding motifs, FSM-GH, ISV-GH, and ITV-GH. The two acidic motifs, EET-GH and EEE-GH, bound at low levels also observed for microsome/beads not permeabilized by detergent. Each histogram bar represents mean ± SEM of transfections with each construct (*n* ≥ 7) with statistical comparisons to APV-GH (**p≤ 0.001, *p≤0.01) or to EET-GH (°°*p* ≤ 0.001). APV, alanine-proline-valine; CEB, Cytosol Extraction Buffer; EEE, glutamic acid–glutamic acid–glutamic acid; EET, glutamic acid–glutamic acid–threonine; ERES, ER exit site; ER-ESCAPE motif, ER-Exit by Soluble Cargo using Amino-terminal Peptide-Encoding motif; FSM, phenylalanine-serine-methionine; GH, growth hormone; HA, hemagglutinin; HEK293A, human embryonic kidney cell line 293; ISV, isoleucine-serine-valine; ITV, isoleucine-threonine-valine; Surf4, surfeit locus protein 4.

True binding-constant analyses require purified proteins and robust reporting systems that produce proportional signals only while the two agents are bound together [29]. We were unable to solubilize and purify Surf4 protein that retained its high-affinity state for APV-GH, an outcome not uncommon for multipass membrane proteins. To obtain an estimate of Surf4’s affinity for GH, microsomes were made from *Surf4*^KO^ cells without and with Surf4 expression by plasmid transfection. As expected if orientation of the microsomes were predominantly in ER’s original configuration (lumen = inside, cytosol = outside), microsomes from Surf4-expressing cells failed to exhibit preferential binding of 400 nM APV-GH (Lane 1) over the same amount of cargo lacking a functional ER-ESCAPE motif, EET-GH (Lane 2) (Fig 10A). Digitonin has long been used to selectively permeabilize cholesterol-enriched plasma membranes. Like other mammalian cells, digitonin-treated HEK293A released cytosolic proteins but only trace levels of soluble lumenal proteins associated with rER and QC domains (e.g., cyclophilin-B, CALR, S4 Fig) because of the low abundance of cholesterol in membranes of these two ER regions. To our knowledge, the amount of cholesterol in purified ERES membranes has not been directly measured, although acute depletion of cholesterol has been reported to cause loss of ER-to-Golgi trafficking of membrane proteins [30]. We found at least a portion of microsomes associated with Surf4 are effectively permeabilized by digitonin such that GH interacted with the necessarily lumenal domain of its cargo receptor. Therefore, additional aliquots of the two microsome preparations were treated with digitonin and assayed for binding. These showed a >5-fold increase in binding of strong ER-ESCAPE motif APV-GH (Fig 10A, Lane 3), while there was no significant increase in binding of EET-GH (Lane 4) by permeabilization. The increased binding of APV-GH to microsomes was dependent on Surf4 because microsomes made from nontransfected *Surf* ^*KO*^ cells had no significant change in binding of either GH construct, with or without digitonin treatment (Lanes 5–8).

To overcome difficulties of working with microsome-based binding assays dependent on multiple centrifugation steps, microsomes were bound to magnetic beads precoated with antibodies to carboxy-terminus (final cytosolic domain) of Surf4. Western blot analysis of microsomes associated with washed beads showed the presence of both Surf4 and ERES/COPII protein, Sec23 (Fig 10B insert), thus supporting microscopy localization of Surf4 in ERES domains. Indeed, there was a dose-dependent increase in binding of 400 nM APV-GH to microsomes as the concentrations of digitonin increased, plateauing at about 30 μg/ml. (Thirty μg/ml concentration of digitonin is similar to that reported by Holden and Horton [29] for Cytosol or Buffer 1, commercially available as Cytosol Extraction Buffer [CEB]) (Fig 10B). CEB was used for all subsequent experiments.

We next tracked the levels of GH retained by digitonin-treated microsomes when equal aliquots of beads were incubated with increasing amounts of APV-GH. GH ELISA results show a sigmoidal increase in APV-GH with half-maximal binding, suggesting a binding constant in the 200–300 nM range. The same concentrations of GH starting with a diacidic tripeptide (EET-GH) showed little retention by permeabilized microsomes (similar to that seen after treatment of permeabilized Surf4^KO^–derived microsomes), verifying apparent lack of affinity by this motif (Fig 10C).

Then, three GH isoforms starting with tripeptides corresponding to modest ability to enhance ER trafficking in vivo (FSM-GH, ISV-GH, and ITV-GH)—as well as a second, poorly trafficked acidic tripeptide, EEE-GH—were compared to the ability of APV-GH and EET-GH to bind to Surf4-associated microsomes. EET-GH and EEE-GH failed to bind Surf4 in permeabilized ERES microsome/beads (Fig 10D). These results correspond well to higher steady-state levels observed in live cell experiments discussed above for GH starting with two or more acidic amino acids. Also, significantly less FSM-GH, ISV-GH, and ITV-GH was retained by the microsomes compared to APV-GH (Fig 10D), results similarly predicted by their steady-state intracellular levels seen earlier in live-cell experiments.

## Discussion

The vesicular nature of ER-to-Golgi trafficking was established by electron microscopy in the 1960s and 1970s [31]. Beginning in 1979 with Novick and Schekman [32], a series of papers identified temperature-sensitive secretion mutants in yeast that opened the door for dissection of underlying biochemical processes. These studies culminated in reports of transport vesicle genesis (COPII) from ER microsomes by addition of purified proteins [33, 34]. The default ER-to-Golgi pathway for soluble cargo proteins is to passively diffuse into the lumen of forming ER exit vesicles and subsequent release into the lumenal fluid of the ERGIC/Golgi (bulk flow). Early pulse-chase experiments of secreted proteins [35] likely underestimated bulk flow rates because of delays caused by chaperone-assisted folding, disulfide bond formation, etc. within rER and QC compartments. Thor and colleagues [36] engineered disulfide-free, glycosylation-free, and rapid, chaperone-independent folding domain of Semliki virus capsid protein as a model protein for bulk flow analysis. This elegant approach resulted in the fastest rate of protein transport reported for mammalian cells. However, the authors were unaware that addition of the HA-tag peptide (YPYDVPDYA) immediately after their leader sequence added a very effective ER-ESCAPE motif. In HEK293A, amino-terminal YPY tripeptide motif effectively trafficked our two most problematical proteins, DSPP and AMELX.

When ER-Golgi bulk flow cannot keep a protein’s concentration below its aggregation potential, use of a cargo receptor becomes necessary. Such cargo receptors should have two basic properties: (1) The binding affinity of a receptor for its cargo must be lower than the concentration at which the protein forms aggregates within the ER. In other words, a cargo receptor cannot effectively bind/traffic a protein until the cargo protein’s local concentration is high enough to significantly interact (≥ K_d_). (2) Receptors must remain in functional excess by either always having an excess of cargo receptors made by the cell and/or by establishing a priority system by which the most problematical proteins can preferentially leave the ER.

Approximately 70% of secreted proteins may acquire cotranslational asparagine-linked (N-linked) oligosaccharide modifications [37]. The discovery that ERGIC-53/LMAN1’s lumenal domain exhibited mannose-binding lectin properties [38] suggested a solution for ER trafficking of many proteins. Indeed, blood-clotting proteins Factor V and Factor VIII are incorrectly trafficked in patients with ERGIC-53/LMAN1 mutations [39]. However, ERGIC-53/LMAN1 was later shown to have a lumenal, soluble helper protein—multiple coagulation factor deficiency protein 2 (MCFD2)—for the clotting factors [40], suggesting that a protein-related motif can be required for this receptor [41]. Alpha1-antitrypsin’s use of ERGIC-53/LMAN1 was also shown to be protein-conformation dependent [42]. Pro-matrix metalloproteinase-9 (proMMP-9) was recently reported to use ERGIC-53/LMAN1 [43]. The presence of a predicted high-affinity ER-ESCAPE motif for proMMP-9 throughout vertebrate evolution (Fig 1) suggests this protease can use either ERGIC-53/LMAN1 or Surf4 for ER trafficking. Therefore, as appealing as use of N-linked oligosaccharides alone for trafficking may be, surprisingly few examples have come to light. Indeed, in our hands, a human GH isoform that is unable to interact with Surf4 was not efficiently trafficked even when a N-linked oligosaccharide was added to this normally nonglycosylated protein.

Direct evidence for receptor-enhanced ER trafficking of soluble cargo using conserved protein/peptide motifs is similarly rare. Belden and Barlowe [23] showed yeast vacuole carboxypeptidase Y (CPY, *PRC1*) and mating factor alpha-1 (mfα-1, *MAT1*) required Erv29p for enhanced ER trafficking. They also showed more pro-mfα-1 trafficked into exit vesicles when purified COPII proteins were added to wild-type ER microsomes, compared to microsomes lacking Erv29p, and that ^35^S-labeled pro-mfα-1 could be crosslinked to Erv29p in microsomes. Caldwell and colleagues [24] also reported Erv29p-enhanced trafficking of CPY as well as a second vacuole protease, PrA (*PEP4*). What is the cargo motif/domain that interacts with Erv29p? *S*. *cerevisiae* mfα-1 has three N-linked oligosaccharides spaced along its 64-amino-acid prepro-domain. Caplan and colleagues [44] showed that successive and combinatorial loss of these modifications resulted in progressive but incomplete loss of hormone secretion. Deletions within this domain of amino acids 23–37 or 29–63 (both left intact the ER-ESCAPE motif at amino acids 20–22) also caused significant but incomplete loss of hormone processing/secretion. Later, Otte and Barlowe [45] focused on a 25-amino-acid domain (residues 29–53) using a series of alanine substitutions to determine the efficiency of trafficking of ^35^S-labeled pro-mfα-1 in the microsome/COPII vesicle in vitro assay. The authors proposed that three spaced (large) hydrophobic amino acids (I39, L42, and V52) together constituted pro-mfα-1’s motif that binds to Erv29p. The authors noted that Erv29p-trafficked protein, pro-CPY, had similar hydrophobic amino acids at same spacing in two of three positions. However, for yeast’s Pry1p and Pry2p, only two of these six primary sequence positions are hydrophobic. Furthermore, in 2009, Rakestraw and colleagues [46] performed mutagenesis on the mfα-1 prepro domain before fusing it to a single-chain antibody sequence and selecting for increased secretion rates (3- to 10-fold over wild type). Four of their eight positive constructs had several mutations that included changes in I39, L42, or V52 (to alanine or a polar/hydrophilic amino acids), with their best construct including both L42S and V52A. More recently, Lin-Cereghino and colleagues [47] reported effects of a series of deletions and alanine substitutions within the mfα-1 prepro domain fused to horseradish peroxidase expressed in *Pichia pastoris*. Deletions of amino acids 30–43 increased the amount of peroxidase activity secreted into the media, although replacement of all 14 amino acids with alanines reduced total secreted activity below that of controls. Therefore, it remains unknown at this time how these three large, hydrophobic amino acids (I39, L42, and V52) in the pro domain that played a role in enhanced trafficking of yeast’s pro-mfα-1 can be extended to other cargo proteins.

Using results of our experiments, we predict that acidic (pI = 4.5) pro-mfα-1 from *S*. *cerevisiae* with its classic Φ-P-Φ tripeptide, APV, has high affinity for Surf4/Erv29p. A limited search for starting tripeptides of pro-mfα-1 in *Saccharomycetales* (S3 Table) shows 36 starting with Φ-P-Φ (24 APV, 8 alanine-proline-isoleucine [API], plus IPV, valine-proline-alanine [VPA], and alanine-proline-alanine [APA]), 4 alanine-proline-threonine [APT], 4 alanine-isoleucine-alanine [AIA], and 1 threonine-alanine-isoleucine [TAI]). One tripeptide our prediction model would suggest has modest to poor binding to ERV29p/Surf4 is pro-mfα-1 for *Candida glabrata* with a QPV motif. This yeast is widely thought to be asexual although recently reported to have an intact mating gene set as well as a pattern of genetic sequences, suggesting a limited sexual cycle [48]. Perhaps the amount of mating factor secreted by this species is low, and the species has lost its requirement for cargo receptor–assisted ER exit. *P*. *kudriavzevii* maintains an mfα-1 starting with an APV motif (S3 Table) but is otherwise lacking the pro-domain. Pro-mfα-1’s motif is similar to two other acidic (pI = 4.3), secreted yeast proteins Pry1p (APA or APV depending on species) and Pry2p (APV), both of which in our experiments trafficked well in wild-type yeast cells but remained predominantly in the ER of *erv29Δ* cells. Lacking a proline or arginine in the number 2 position, the starting tripeptides of yeast proteins CPY (isoleucine-serine-leucine [ISL] ≈ ISV-GH) and PrA (KVH-GH) had more modest binding in our GH assay than APV-GH, but both still had sufficient affinity for the receptor to explain the reported low ER steady-state levels in wild-type yeast when compared to *erv29Δ* cells [23, 24]. Invertase (starting with serine-methionine-threonine [SMT] and repeatedly reported not to use Erv29p) we predict to be a weak or nonbinding cargo protein for Erv29p. One indication that proline in amino-terminal number 2 position could be related to trafficking of at least Golgi/ER-resident proteins in higher eukaryotes was in 2009 when Tsukumo and colleagues [49] used a series of alanine substitutions within the first nine amino acids (after removal of signal peptide) of nucleobindin-1 (*NUCB1*). They showed the P28A mutant of this normally Golgi-resident protein had increased localization in the ER, decreased Golgi-related modifications, and decreased secretion. They also reported three other ER/Golgi-resident proteins (reticulocalbin-1, calumenin, and 45 kDa Ca^2+^-binding protein [Cab45]) were more highly localized to ER when number 2 prolines were changed to alanines.

In 2001, Caldwell and colleagues [24] noted that Surf4 was a human homolog of Erv29p sharing approximately 30% amino acid identity (S3 Fig). *Surf4* is one of four genes closely spaced in the Surfeit cluster of higher eukaryotic species [26]. These genes have no known sequence relationships and were proposed to be housekeeping genes because of their expression in a number of differentiated mouse cell lines as well as the presence of unmethylated, CpG-rich islands in their 5′ domains [25]. Human Surf4 protein sequence was first described in 1995 by Reeves and Fried [27] as a 30 kDa membrane protein with up to seven predicted transmembrane domains and an ER-like cytolocalization consistent with a proposed dilysine ER-retrieval motif near the carboxy-terminal end of the protein. In 2008, Mitrovic and colleagues [50] showed HeLa cell Surf4 predominantly colocalized with ERGIC-53-associated structures with some costaining with early Golgi domains. The authors also verified that Surf4 was likely an ER-Golgi-ER cycling protein by replacing Surf4’s three near-carboxy-terminal lysines with serines, causing the protein to accumulate in the Golgi. We found similar immunocytological localization although with much more ERES expression and weblike structures around ERES for both wild-type and HA-tagged Surf4 in HEK293A cells. Our results extended these observations to note that Surf4 was generally not abundant in rER or QC domains. We support the COPI-retrieval motif hypothesis by showing mutation of two of three carboxy-terminal lysines to alanines caused both a significant increase *cis*-Golgi accumulation and failure to rescue efficient trafficking of specific cargo in *Surf4*^*KO*^ cells. Mitrovic and colleagues also noted that siRNA knockdown of Surf4 to <10% of normal had no effect on the ER-associated degradation (ERAD) of the Z mutant (E366K) of alpha1-antitrypsin, leading them to argue against Surf4 functioning in higher eukaryotic species (as Erv29p was proposed by Caldwell and colleagues [24] to do for yeast) by trafficking misfolded proteins into the Golgi for subsequent degradation. While we have no comment on the ultimate role Surf4 may play in ERAD of misfolded proteins, it is noteworthy that for Caldwell and colleagues’ misfolded proteins, CPY* and PrA*, the proteins both apparently started with their functional ER-ESCAPE motifs discussed in the above paragraph, possibly retaining their ability to be trafficked out of yeast ER by Erv29p. The mutant α1-antitrypsin construct, however, apparently retained its nonbinding diacidic-starting tripeptide (glutamic acid–aspartate acid–proline [EDP]), rendering it unlikely to interact with Surf4 in either its native or misfolded forms. To our knowledge, no one has directly shown that the homolog of Erv29p, Surf4, is a functioning ER cargo receptor enabling more efficient trafficking of soluble proteins to the ERGIC/Golgi in higher eukaryotic cells. Indeed, Mitrovic and colleagues [50] noted that knockdown of Surf4 by siRNA in HeLa cells caused no significant changes in total ^35^S-methionine-labeled protein secretion.

After showing yeast Erv29p used the ER-ESCAPE motif to enhance trafficking of acidic proteins out of the ER, we next used CRISPR/Cas9 technology to delete the human homolog, *SURF4*, in HEK293A cells. The failed trafficking of our two most problematical matrix proteins, the Ca^2+^-binding DSPP and self-assembling AMELX, in *Surf4*^*KO*^ cells was rescued by coexpression of Surf4 for wild-type cargo proteins but not when the cargo included a motif-damaging acidic amino acid (IPD-DSPP and EPL- AMELX). Because Erv29p rescued ER trafficking of cargo in the *Surf4*^KO^ cells, it appears that many major functions of this receptor are conserved across all eukaryotes. The ER-ESCAPE motif for Surf4 is limited to the first three amino-terminal amino acids because introduction of bulky N-linked oligosaccharide to the fourth amino acid of GH did not hinder enhanced ER trafficking. This same cotranslational modification is naturally found on the fourth amino acid of yeast’s classic Erv29p cargo protein, pro-mfα-1 [51, 52], and may ensure the amino-terminus is readily available for receptor binding. Based on this work, use of a high-affinity ER-ESCAPE motif (IPV, RSV, etc.) directly after a strong leader sequence is highly recommended for production of secreted recombinant proteins in eukaryotic cell expression systems. When an amino-terminal antibody tag is required, an HA-tag directly after the leader sequence offers the benefit of a very strong ER-ESCAPE motif, YPY.

The Golgi KDEL receptor binds the carboxy-terminal tetrapeptide KDEL (sometimes, HDEL) of escaped ER-resident soluble proteins such as chaperones and protein-modifying enzymes, returning them to the ER [53]. Our estimated binding constant of Surf4 for a strong Φ-P-Φ ER-ESCAPE motif was 200–300 nM, an affinity similar to 78–200 nM reported for the KDEL receptor [53]. For the energetically valuable process of scavenging escaped chaperones, it makes sense such proteins would have a single or narrow range of high-affinity motifs for receptor binding. In contrast, a range of peptide motifs with different binding affinities is of benefit for prioritizing the ordered ER exit of problematic proteins.

**Fig 11:**
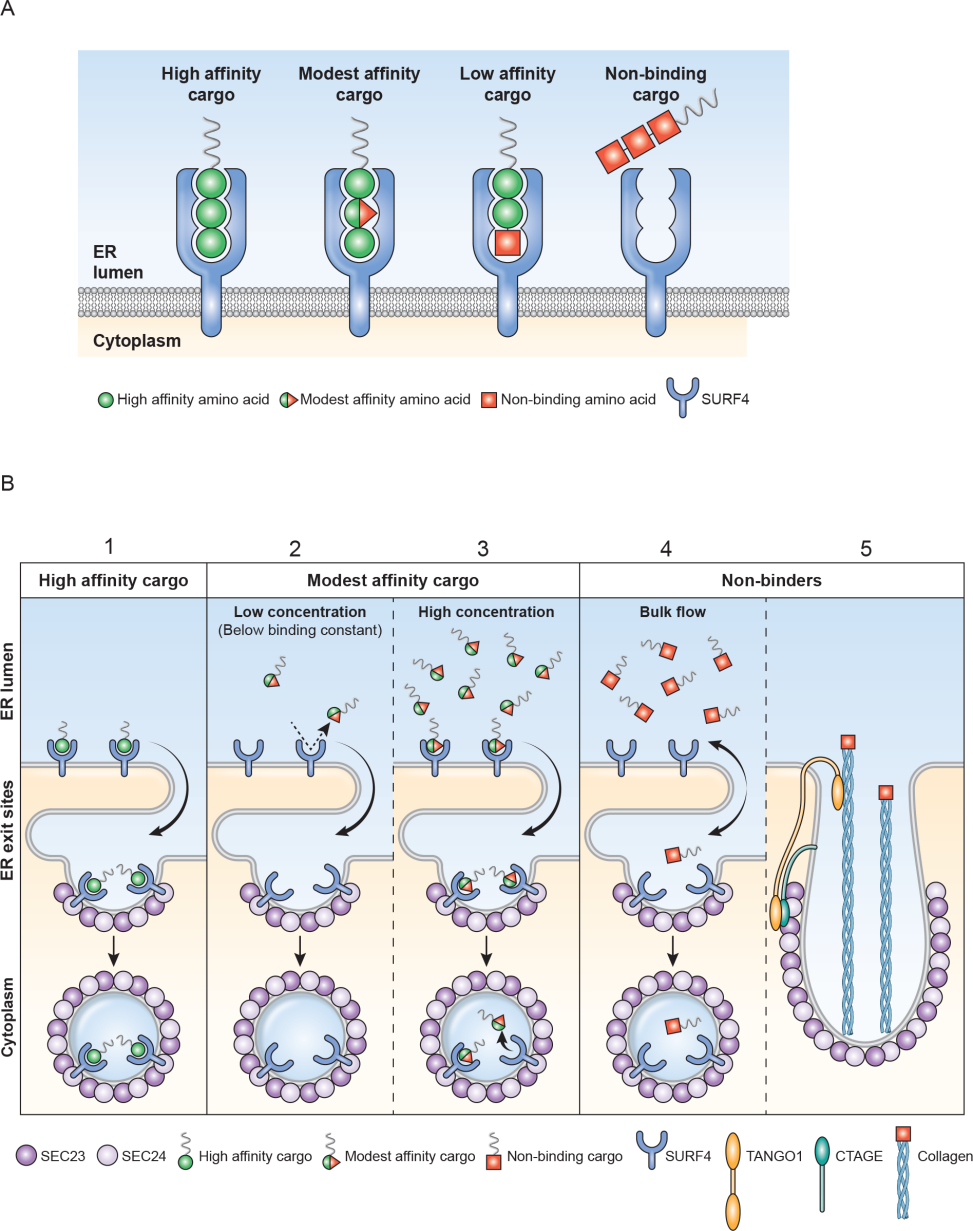
Model illustrating interaction of ER-ESCAPE motifs with high, modest, and no affinity for Surf4/Erv29p. (A) A green ball amino acid in spherical pocket denotes highest contribution of that residue to binding affinity such as a proline in number 2 position or a hydrophobic residue in position 1 (amino-terminus) or 3. Green half-ball plus red pyramid represents lower affinity interaction for that amino acid (e.g., serine in position 2), while the red cube denotes a negative contribution to binding affinity (e.g., acidic amino acid in position 1). High-affinity cargo present high-affinity contributions in all three positions, while modest- to low-affinity tripeptides have at least one mismatch. Nonbinding proteins such as chaperones or fibrillar collagens have two or three completely mismatching amino acids. (B) (1) High-affinity cargo (e.g., IPV) are bound to Surf4/Erv29p and exit ER before they accumulate to aggregate-forming concentrations. (2) Cargo with more modest ER-ESCAPE motifs (e.g., FSM) do not significantly bind to cargo receptor until (3) they accumulate to levels ≥ their binding constant. Only at that point do they remain bound long enough to remain in COPII vesicle at levels significantly greater than bulk flow. (4) illustrates cargo starting with nonbinding amino-terminal tripeptides (e.g., QEE) cannot exit ER more efficiently than their concentration in ER lumenal fluid in equilibrium with the small amount of exit vesicle fluid (bulk flow). (5) Fibrillar collagens are too large for standard COPII exit vesicles and must use more voluminous TANGO1/cTAGE5-associated exit vesicles. Large fibrillar collagens often start with nonbinding motif (e.g., QEE) to keep them from binding Surf4 and partially entering smaller COPII vesicles. COPII, coat protein complex II; ER, endoplasmic reticulum; ER-ESCAPE motif, ER-Exit by Soluble Cargo using Amino-terminal Peptide-Encoding motif; FSM, phenylalanine-serine-methionine; IPV, isoleucine-proline-valine; QEE, glutamine–glutamic acid–glutamic acid; Surf4, surfeit locus protein 1; TANGO1, transport and Golgi organization 1.

Our current model consists of several points. First, each of the three amino-terminal amino acids contribute to affinity of a cargo protein for the binding pocket located in lumenal domain(s) of Surf4/Erv29p (Fig 11A). Second, each cargo can bind only when its local concentration is bigger than or equal to its binding constant (Fig 11B). Cargo with high affinity may be quickly removed from the ER lumenal fluid, while proteins with more modest affinity ER-ESCAPE motifs bind and release more frequently while being processed for entry into the COPII vesicle. Some cargo (e.g., collagens too large to fit into standard-sized exit vesicles) start with tripeptides with no affinity for Surf4 and are directed to specific exit vesicles lacking Surf4. Small soluble cargo proteins (e.g., chaperones) with no affinity for Surf4 exit the ER only by diffusion into the lumenal fluid of vesicles (bulk flow) (Fig 11).

**Fig 12:**
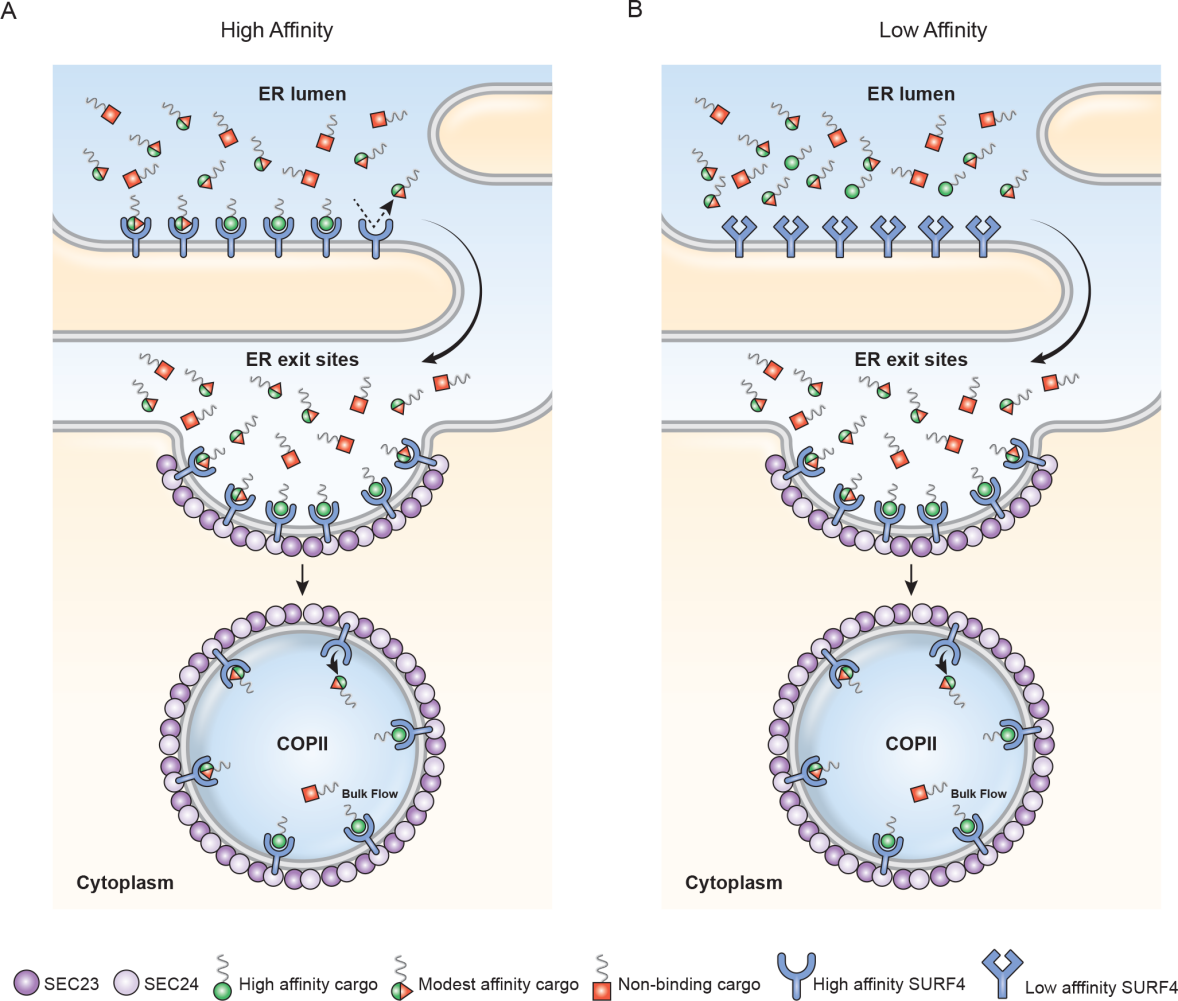
Exit of highest affinity cargo is prioritized in ERES lacking an excess of Surf4/Erv29p. Cargo receptors must have a high-affinity conformation to bind cargo in the ER and a low-affinity conformation to release cargo in fully formed exit vesicle or upon fusing with ERGIC/Golgi. High-affinity panel (A) illustrates a model whereby cargo receptors in the vicinity of ERESs have the ability to bind cargo before physically entering COPII vesicle, while the low-affinity panel (B) represents an alternative model in which the receptor is in its low-affinity state until interacting with elements of COPII vesicle (e.g., Sec24). In both cases, when there is an excess of cargo for local population of receptors, high-affinity cargo occupies available receptors, while lower-affinity cargo continues to build in concentration. This aids in keeping the most problematical proteins below their aggregation concentrations. Similarly, modest-affinity cargo, when their concentration becomes ≥ binding constant, occupy any available receptors before cargo with still-lower-affinity ER-ESCAPE motifs. This process delays aggregate formation until cargo receptors can be brought into balance with local/total cargo loading. Nonbinding cargo continue to exit solely by diffusion/equilibrium between fluids of COPII vesicle and ERES lumen (bulk flow). COPII, coat protein complex II; ER, endoplasmic reticulum; ERES, ER exit site; ER-ESCAPE motif, ER-Exit by Soluble Cargo using Amino-terminal Peptide-Encoding motif; ERGIC, ER-Golgi intermediate compartment; Erv29p, ER-derived vesicles protein 29; Surf4, surfeit locus protein 4.

The chaperone CALR is reported to be secreted to some extent into the extracellular environment [54, 55] perhaps due, in part, to its low- but positive-affinity ER trafficking by Surf4. However, a more interesting consequence of CALR having a low-affinity ER-ESCAPE motif (Fig 1) may be in controlling binding of Surf4 cargo in the rER and QC regions. CALR’s high concentration in the rER/QC domains may outcompete most soluble cargo proteins for newly synthesized Surf4 such that the receptor does not interfere with protein folding. However, low levels of Surf4 in the rER and QC domains may bind high-affinity motifs of the most problematic proteins (e.g., DSPP), even in the presence of high CALR levels, and keep cargo from aggregating within rER/QC lumen. Furthermore, disordered proteins like DSPP and OPN lack appropriate hydrophobic domains used in proposed “ratcheting” mechanisms whereby BiP/GRP78 binds to early hydrophobic folding domains of cargo as they are translocated into the rER and keeps the growing protein from diffusing back out of translocon pores and into the cytosol [56, 57]. In this model, Surf4 could substitute for BiP by binding to the high-affinity ER-ESCAPE motif on these disordered proteins early in translocation. Our Surf4 siRNA experiments support the concept that when Surf4/Erv29p is not locally in excess, cargo with higher-affinity motifs (i.e., those most likely to form damaging aggregates) are preferentially trafficked out of the ER (Fig 12).

Surf4/Erv29p are multipass proteins, with the number of transmembrane domains predicted/reported ranging from four to seven [24, 27, 58]. Surf4/Erv29p’s tripeptide-binding pocket may result from cooperative three-dimensional alignment of two or more lumenal domains. By analogy, binding of extracellular ligands causes some plasma membrane–associated G-protein-coupled seven-transmembrane receptors to undergo conformational changes, resulting in corresponding realignment of cytosolic domains and binding/activation of G-alpha proteins [59]. Thus, binding of cargo to Surf4/Erv29p could result in conformational changes in its transmembrane domains that reorient cytosolic domains and enable interaction with COPII proteins such as Sec24 (Fig 12A). Of course, the opposite is an equally attractive model whereby only after Surf4/Erv29p interacts with a COPII-associated protein such as Sec24 does formation of the high-affinity, ER-ESCAPE motif–binding pocket occur (Fig 12B). Only then could ER-ESCAPE motif–presenting cargo proteins bind to Surf4/Erv29p and preferentially join developing exit vesicles. Future studies defining binding and functional domains of Surf4/Erv29p will help us understand the order in which cargo binding and COPII vesicle formation occur, as well as how the receptor releases its cargo after leaving the ERES.

In summary, a cell’s ER-to-ERGIC/Golgi bulk flow process can be insufficient for keeping specific abundantly secreted proteins’ ER concentration sufficiently low to prevent premature or inappropriate aggregate formation. We have shown that functionally conserved ERES transmembrane protein Surf4 enhances trafficking efficiency of several problematic cargo proteins by binding to their amino-terminal tripeptides. The combinatorial chemical composition of the amino-terminal three amino acids results in different Surf4-binding affinities and therefore the corresponding steady-state levels of each cargo within the ER lumen. The lower the protein’s concentration that can result in aggregate formation, the higher affinity the starting tripeptide evolutionally acquired and maintained. Generally, Φ-P-Φ tripeptides (with arginine substitutions in any position) result in high-affinity binding and associated low steady-state ER levels. In contrast, soluble proteins that should not enter standard COPII vesicles (chaperones and large fibrillar collagens) generally have amino-terminal tripeptides with no Surf4 affinity by including glutamine or acidic amino acids in the motif. Whenever local Surf4/Erv29p levels become limiting, the differing binding affinities automatically prioritize exit of the high-affinity cargo over proteins with lower-affinity motifs. At this time, interactions of the 8,000 possible permutations of three starting amino acids with Surf4 are too complex to be unfailingly predicted by our proposed ER-ESCAPE-motif formula (particularly for modest-/low-affinity motifs). However, using a combination of the current formula, as well as verification of the tripeptide’s conservation among the homologous proteins of many species, can be a useful indication of the strength of protein–Surf4 (Erv29p) interactions. Future high-throughput binding experiments of all 8,000 tripeptides in conjunction with protein database bioinformatics research of all known ER-translocated soluble proteins (with solution-available amino-termini) will result in more comprehensive prediction models.

## Materials and methods

### Cell culture and transfection

HEK293A (Thermo Fisher Scientific) and *Surf4*^KO^ HEK293A cells (see following subsection, Generation of *Surf4*^KO^ HEK293A cells) were maintained in Dulbecco's Modified Eagle Medium (DMEM, Thermo Fisher Scientific) supplemented with 10% fetal bovine serum (Sigma), GlutaMAX (2 mM, Thermo Fisher Scientific), penicillin 100 (IU/mL), and streptomycin (100μg/mL). Expression constructs were transfected into cells using Lipofectamine 2000 reagent (Thermo Fisher Scientific). Briefly, 5×10^5^ cells/well were plated into 6-well plates. After overnight culture (60%–80% confluent), cells were transfected with 2μg of plasmid DNA preincubated with 4μL Lipofectamine 2000 in Opti-MEM (Thermo Fisher Scientific). Transient expression of proteins was analyzed 16–24 hr posttransfection as noted.

### Generation of *Surf4*^KO^ HEK293A cells

*Surf4*^KO^ HEK293A cells were generated under contract by Applied StemCell using CRISPR/Cas9 technology by targeting two sites in exon-3 of human *Surf4*, with two independent gRNA (S2A Fig). The genomic deletion was confirmed in three independent single-cell clones—clone 5.3, clone 5.5, and clone 5.19—by PCR (S2B Fig) and sequencing of TOPO clones (S2C Fig). After preliminary confirmation of similar results by all three clones, clone 5.3 was selected for subsequent experiments. KO of Surf4 protein was confirmed by immunoblot using affinity-purified rabbit antibody to Surf4 (S4CT, against carboxy-terminal peptide: CALGPGGVSMDEKKKEW).

### Expression constructs

Plasmid vectors for wild-type and mutant DSPP (including 6xFLAG-tag, when noted) were previously described [5]. Full-length human *AMELX* (variant-3) was amplified by high-fidelity PCR from cDNA (OriGene Technologies) and subcloned into Gateway pENTR/D-TOPO (Thermo Fisher Scientific). AMELX, variant-1, the most abundant variant in human enamel, was generated by deleting exon-4 of variant-3 and used in all experiments. Both Myc-tag at carboxy-terminus and first tripeptide mutations EPL, ISV, and FSM were introduced in AMELX by Q5 Site-Directed Mutagenesis (Q5 Site-Directed Mutagenesis Kit; NEB) using NEB’s web-based oligonucleotide design program. Full-length human growth hormone-1 (hGH) was amplified by high-fidelity PCR of cDNA (TransOMIC Technologies) and subcloned into Gateway pENTR/D-TOPO. First tripeptide mutations and fourth position NTT N-linked oligosaccharide motif mutations were introduced by Q5 Site-Directed Mutagenesis. Full-length human *Surf4* (isoform-1) was obtained by high-fidelity PCR of cDNA (TransOMIC Technologies) and subcloned into Gateway pENTR/D-TOPO. HA-tag was introduced to amino-terminus of *Surf4* immediately after start methionine by Q5 Site-Directed Mutagenesis. Full-length *PRY1*, *PRY2*, and *ERV29* genes in *S*. *cerevisiae* were amplified by high-fidelity PCR of genomic DNA and cloned into Gateway pENTR/D-TOPO. Amino-terminal HA-tag was introduced to plasmid for *ERV29* after Ser37 by Q5 Site-Directed Mutagenesis. Amino-terminal 2×HA-tag and carboxy terminal Myc-tag were introduced by Q5 Site-Directed Mutagenesis to plasmids for *PRY1* and *PRY2*. Then, 2×HA-tag was inserted after Thr27 for Pry1p and Thr26 for Pry2p. APD-Pry1 and APD-Pry2 were introduced by Q5 Site-Directed Mutagenesis to 2XHA-tagged plasmids for *PRY1* and *PRY2*. All pENTR clones were shuttled into Gateway pT-Rex-DEST 30 eukaryotic expression vector (DSPP, AMELX, GH, Surf4, and ERV29) or Gateway pYES-Dest52 *S*. *cerevisiae* expression vector (*PRY1*, *PRY2*, DSPP) (Thermo Fisher Scientific), or pAG425GPD-ccdB (a gift from Susan Lindquist, Addgene plasmid # 14154) for *ERV29*. Plasmids were confirmed in every case by Sanger DNA sequencing.

### Yeast media and strains

The *S*. *cerevisiae* strains are listed in S4 Table. Transformation was performed using *S*.*c*. EasyComp kit (Thermo Fisher Scientific). Isolated colonies were selected and grown in 2% (w/v) raffinose and synthetic drop-out medium (without uracil; without both uracil and leucine; or without histidine, leucine, tryptophan, and uracil) depending on plasmid-selection needed (S5 Table) for approximately 48 hr until OD_600_ ≈ 2–3. Cultures were diluted to OD_600_ = 0.4 and induced in same drop-out medium containing 2% (w/v) galactose and 1% (w/v) raffinose for 5 hr before harvesting. Cells were lysed with Y-PER Yeast Protein Extraction Reagent (Thermo Fisher Scientific). Total protein concentration was determined by Pierce BCA protein assay (Thermo Fisher Scientific). Conditioned media were concentrated by Amicon Ultra-4 Centrifugal Filter Units, 10 kDa cutoff (Millipore Sigma). All yeast cultures were grown at 30°C with shaking at 200 rpm.

### Immunoblotting

Rabbit antiserum against DSP portion of mouse DSPP (LF-153) was as described [60]. Other primary antibodies used for western analysis include mouse anti-Flag M2 (1:1,000, Sigma, Cat #: F1804), mouse anti-Myc (1:1,000, Cell Signaling Tech. Cat #: 2276), mouse anti-β-Actin (1:1,000, Sigma, Cat #: A1978), rabbit anti-HA (1:1,000, Cell Signaling Tech. Cat #: 3724), goat anti-hGH (1:500, Santa Cruz Biotech. Cat #: sc-10365), goat anti-SEC23 (1:200, Abcam, Cat #: AB99522), mouse anti-CALR (FMC75) (1:1,000, Calbiochem, Cat #: 208912), rabbit anti-cyclophilin B (1:1,000, Abcam, Cat #: ab16045) and custom-prepared and affinity-purified rabbit anti-S4CT (0.5 μg/ml). Secondary antibodies include IRDye 680 donkey anti-rabbit IgG, IRDye 800 donkey anti-mouse IgG, and IRDye 680 donkey anti-goat IgG (1:20,000, LI-COR Biosciences).

#### Whole-cell lysates

Cells were harvested and lysed in NP-40 lysis buffer (1% Nonidet P-40 [Calbiochem], 50 mM Tris-HCl [pH 8.0], 100 mM NaCl, 2 mM EDTA, and cOmplete EDTA-free protease inhibitor cocktail [Roche Holding AG]). DM lysis buffer (0.5% n-Dodecyl-α-D-Maltopyranoside [Anatrace], 50 mM Tris-HCl [pH 8.0], 100 mM NaCl, 2 mM EDTA and cOmplete EDTA-free protease inhibitor cocktail) was used to solubilize Surf4. Total protein concentration was determined by Pierce BCA protein kit (Thermo Fisher Scientific). Media were concentrated by Amicon Ultra-4 Centrifugal Filter Units, 10 kDa cutoff. Lysates or concentrated conditioned media were added to NuPAGE LDS sample buffer (Thermo Fisher Scientific) and incubated at 70°C for 10 min before electrophoresis. Surf4 preparations were treated at RT overnight in NuPAGE LDS sample buffer containing 4 M urea. Equal amounts of total protein of cell lysates or conditioned media volumes were electrophoresed on NuPAGE 4%–12% Bis-Tris PAGE gels in MOPS Buffer (Thermo Fisher Scientific) and transferred onto Immunobilon-FL membranes (Millpore). PageRuler Prestained NIR Protein Ladder is used as MW marker (Thermo Fisher Scientific). Membranes were blocked in Odyssey Blocking Buffer (LI-COR Biosciences), followed by incubation with indicated antibodies overnight in PBS with 1% Tween 20 (PBS-T). After 4× 5 min washes in PBS-T, blots were incubated for 1 hr at RT with noted species-specific IRDye-conjugated secondary antibody in PBS-T plus 0.02% SDS. After 3 final washes, blots were quantified by densitometry using LI-COR Odyssey infrared imaging system.

### RT-PCR analysis of XBP-1 mRNA splicing

Total RNA was extracted from HEK293A cells 52 hr after transfection or 5 hr of tunicamycin (2 μg/mL) treatment using an RNeasy Kit (Qiagen). Aliquots of 1 μg of total RNA were reverse-transcribed with SuperScript III First-Strand Synthesis System, PCR-amplified with Platinum Taq DNA polymerase (both from Thermo Fisher Scientific) using primers corresponding to nucleotides 374–395, (5′-GCCTTGTAGTTGAGAACCAGGA-3′) and 623–642 (5′-TGACTGGGTCCAAGTTGTCC-3′) of human XBP-1, resolved on a 6% TBE gel.

### Deglycosylation and protein analysis

Indicated cell lysates and cultured media containing noted N-linked glycosylated GH proteins were treated with Endoglycosidase H or PNGase F (New England BioLabs). Briefly, 9 μl of sample containing either 3 μg of lysate protein or 6% of concentrated media was denatured with 1 μL 10×Glycoprotein Denaturing buffer at 95°C for 10 min. Then, 10×GlycoBuffer-3 plus 250 units of Endo H, or 10× GlycoBuffer-2 + NP-40 and 250 units of PNGase F, was added and incubated at 37°C for 2 hr.

### Immunocytochemistry

Primary antibodies used include mouse anti-HA (1:200, Abcam, Cat #: AB18181), rabbit anti-HA (1:200, Cell Signaling Tech. Cat #: 3724), goat anti-SEC23 (1:50, Abcam, Cat #: AB99522), rabbit anti-Giantin (1:1,000, BioLegend, Cat #: 924302), mouse anti-calnexin (1:200, Thermo Fisher Scientific, Cat #: MA3-027), mouse anti-ERGIC-53 (1:100, Enzo, Cat #: ENZ-ABS300-0100), goat anti-SEC61α (1:100, Santa Cruz Biotech. Cat #: sc-12322), or rabbit anti-S4CT (2.5 μg/ml) antibodies. Secondary antibodies (1:500, Thermo Fisher Scientific) used in these experiments include Alexa Fluor 568-conjugated goat anti-mouse, Alexa Fluor 488-conjugated goat anti-rabbit, Alexa Fluor 568-conjugated donkey anti-goat, Alexa Fluor 488-conjugated donkey anti-rabbit, Alexa Fluor 568-conjugated donkey anti-goat, Alexa Fluor 488-conjugated donkey anti-mouse, and Alexa Fluor 488-conjugated with Alexa Fluor 568-conjugated goat anti-rabbit.

Eight-well Nunc Lab-Tek II-CC2 Chamber Slide was pretreated for 1 hr with 500 μl of 15 μg/ml fibronectin (Sigma) per well before brief washing with PBS. Wild-type or *Surf4*^KO^ HEK293A cells were plated at 40,000 cells/well. After 15 hr, cells in Opti-MEM were transfected with 0.2μg of plasmid DNA prebound to 0.4μL Lipofectamine-2000. Eighteen hr posttransfection, cells were fixed for 10 min using 4% paraformaldehyde/PBS and then permeabilized with 0.2% saponin (Sigma) in LI-COR Odyssey Blocking Buffer-PBS for 2 hr at RT. Primary antibody was diluted in blocking buffer containing 0.2% saponin. Cells were incubated in primary antibody overnight at 4°C, washed 3× in PBS, and incubated for 45 min at RT with indicated secondary antibodies in PBS. Cells were washed 3x in PBS and mounted with VECTASHIELD antifade mounting medium with DAPI (Vector Laboratories). Images were obtained using a Zeiss LSM 780 Confocal microscope (63×/1.40 oil objective, Carl Zeiss) or Zeiss Axio Imager Z1 with Apotome 2 (single Z stack slice, 63×/1.40 oil or 100×/1.40 oil objective, Carl Zeiss). Images were analyzed using Zeiss’ Zen software.

### SEAP detection by Quanti-Blue assay

HEK293A and *Surf4*^KO^ cells were transfected as describe above with pSELECT -zeo-SEAP (InvivoGen). Twenty-two hr posttransfection, 5μL conditioned media was assayed for SEAP activity using QUANTI-Blue (InvivoGen).

### Luciferase-tagged LPO secretion detection by BioLux Gaussia Luciferase Assay

pGAUS3 plasmid for Gaussia luciferase-tagged LPO (VL5) was a gift from Peter Burbelo, NIDCR/NIH. First tripeptide mutations RSV and IPV were introduced in LPO by Q5 Site-Directed Mutagenesis (Q5 Site-Directed Mutagenesis Kit; NEB). HEK293A and *Surf4*^KO^ cells were separately transfected with wild-type LPO (QTT) and two first tripeptide mutations (RSV-LPO and IPV-LPO). Cells and conditioned media were harvested 22 hr posttransfection. Washed cells were lysed in NP-40 lysis buffer containing cOmplete EDTA-free protease inhibitor cocktail. Total protein concentration was determined by Pierce BCA protein assay kit. Luciferase activity was determined using 5 μl of conditioned media or 5 μl cell lysate by BioLux Gaussia Luciferase Assay kit (NEB) following assay protocol II using Berthold Technologies CentroXS^3^ LB960 Luminometer. The Luciferase activities from cell lysates were normalized to Luciferase units/mg protein.

### DSPP and AMELX intracellular aggregation detection

HEK293A *Surf4*^*KO*^ cells at about 70% confluency in 6-well cell plates were transfected with IPV-DSPP expression plasmid (as above) and harvested 24 hr later. Cell pellet was treated with CEB from Subcellular Protein Fractionation Kit (Thermo Fisher Scientific) in +/− 10 mM CaCl_2_ on ice for 10 min and centrifuged at >100,000 x g for 10 min (A100/18 rotor in Beckman Airfuge). Ten percent of concentrated supernatant and 10% of pellet were analyzed by western blot. HEK293A *Surf4*^*KO*^ cells were transfected with MPL-AMELX expression plasmid (as above) and harvested 24 hr later. Cell pellet was treated with MEB from Subcellular Protein Fractionation Kit on ice for 10 min and centrifuged at >100,000 x g for 10 min (A100/18 rotor in Beckman Airfuge). Ten percent of concentrated supernatant and 10% of pellet were analyzed by western blot.

### hGH ELISA for transfected HEK293A cell lysates

HEK293A cells were separately transfected with 60 expression plasmids encoding noted changes in post-leader sequence tripeptide of hGH. Each experiment included both positive (IPV-GH) and negative (EET-GH) trafficking controls. Cells were harvested 18 hr posttransfection with 0.5% trypsin (3 min); the reaction was stopped by washing with PBS, followed by single-cell suspension in 10% fetal bovine serum in DMEM. Cells were briefly washed in PBS, pelleted, and lysed in NP-40 lysis buffer containing cOmplete EDTA-free protease inhibitor cocktail. Total protein concentration was determined by Pierce BCA protein assay. Concentration of GH in cell lysates was determined in triplicate in each experiment by hGH ELISA (IBL America, Cat #: IB19101). GH concentrations from all cell lysates were calculated by standard curve (GraphPad Prism 7, Graphpad Software) and normalized to standard total protein concentration (ng GH/mg protein). The amount of GH present in 59 non-IPV variants of starting tripeptides was normalized to concentrations of control levels of IPV-GH associated with that transfection series. Data represent means ± SEM of at least 3 transfection experiments for each GH variant. (Means of triplicate hGH ELISA analyses were used for each extract).

### siRNA transfection

Silencer Select predesigned siRNA for human Surf4 and *Silencer* Select Negative Control No. 1 siRNA. Surf4 was knocked down using 5 nM of both 5′-AGAAUGAUGCAGCAUUAAAtt-3′ as sense and 5′-UUUAAUACUGCAUCAUUCUtt-3′ as antisense oligo with Lipofectamine RNAiMAX transfection reagent (all from Thermo Fisher Scientific). ITVNTT-GH and APV-GH were cotransfected 36 hr later into same HEK293A cells. Cells were harvested 18 hr later and processed for Surf4 and GH western blot following procedures noted above.

### Microsome preparation and GH-binding assay

Tripeptide variant GH proteins (APV, ITV, ISV, FSM, EEE, and EET) were prepared from conditioned media collected 24 hr after transfection with corresponding GH plasmids into HEK293A cells. Concentrations were measured by hGH ELISA and adjusted to noted concentrations by dilution into KHMC buffer.

#### Crude microsome preparation

*Surf4*^KO^ cells, either untreated or transfected for 24 hr with 12 μg of HA-Surf4 expression plasmid preincubated with 24 μl Lipofectamine 2000 in Opti-MEM, were released from 100 mm culture dishes, suspended in 6 ml of ice-cold hypotonic buffer (5 mM EDTA, 20 mM HEPES, pH 8.0, cOmplete EDTA-free protease inhibitor cocktail), and homogenized with a 7 mL Dounce glass homogenizer using a tight pestle (Wheaton Industries) until >90% of cells were ruptured. Homogenates were cleared of large debris by centrifugation at 6,000 ×g (10 min, supernatant) and then 60 min at 100,000 ×g (pellets). The microsomes were suspended in KHMC buffer by repeated pipetting. All above steps were conducted on ice or at 4°C. Half of the microsome preparation was pelleted and treated with CEB from Subcellular Protein Fractionation Kit for 30 min on ice before resuspension to starting volume with KHMC buffer. Microsomes, plus or minus CEB treatment, were equally aliquoted into separate experimental tubes and centrifuged at >100,000 x g for 10 min (A100/18 rotor in Beckman Airfuge). Pellets were suspended in 175 μl of 400 nM APV-GH or EET-GH media in KHMC buffer for 1 hr on ice. After >100,000 xg centrifugation (10 min), pellets were suspended for 10 min in 1 mg/ml APVNTTGGGC peptide to release bound GH. After final 10 min >100,000 xg centrifugation, amount of GH in supernatant was assayed using hGH ELISA kit.

#### ERES-associated microsome preparation using Surf4 antibody-coated magnetic beads

HEK293A cells were transfected with HA-Surf4 expression plasmid as above. Homogenates prepared by methods described above were centrifuged at 6,000 xg for 10 min to clear cell debris and supernatants collected for following studies. Surf4-enriched microsomes were prepared using 10 μg affinity-purified rabbit anti-Surf4-CT prebound to each 50 μl aliquot of magnetic Protein G-Dynabeads (Thermo Fisher Scientific). Briefly, microsome-containing supernatants (containing protease inhibitors) were gently mixed by rotation with washed anti-Surf4-Dynabeads for 1 hr at RT. Dynabeads were recovered using a magnetic particle separator and washed 3 times in KHMC buffer. Where noted, aliquoted microsome/beads were treated with CEB or 10–40 μg/ml digitonin (SIGMA, Cat # D141) in KHMC buffer for 30 min at RT. Final microsome/beads were quickly washed 3 times with KHMC buffer and recovered using a magnetic particle separator. For estimation of APV-GH binding constant, equal aliquots (in triplicate) of CEB-treated microsome-Dynabeads were incubated/tumbled with 0 nM, 100 nM, 200 nM, 300 nM, 400 nM, or 800 nM of APV-GH or EET-GH for 1 hr at RT. For relative binding levels of APV-GH, ITV-GH, ISV-GH, FSM-GH, EEE-GH, or EET-GH to microsome/beads, 400 nm of each GH variant was added to equal aliquots (in triplicate) of CEB-permeabilized microsome-Dynabeads in KHMC buffer and incubated for 1 hr with RT tumbling. (Note that one triplicate experiment involved incubation of 400 nM APV-GH with an equal aliquot of microsome/beads not treated with CEB.) Microsome/beads were then briefly washed twice with KHMC buffer and GH eluted by APVNTTGGGC peptide (1 mg/ml) for quantification by ELISA assay.

### Statistical analysis

Statistical analyses were performed using GraphPad Prism 7 (Graphpad Software). One-way ANOVA analysis was carried out at IBM SPSS software. Results are expressed as mean±SEM.

The numerical data used in all figures are included in S1 Data.

## Supporting information

S1 Data Excel spreadsheet containing original numerical data and statistical analysis for Figs panels 6B, 6H, 8A, 9D, 10A, 10B, 10C and 10D. (XLSX)

S1 Fig Among 12 candidate yeast ER exit vesicle proteins deleted, only loss of Erv29p resulted in predicted loss in trafficking of both wild-type IPV-DSPP and ER-ESCAPE motif mutant IPD-DSPP. Immunoblot of wild-type DSPP^Flag^ (IPV) or mutant (IPD) conditioned media in the indicated 12 ER exit vesicle-rich, transmembrane protein knockout strains. Cells were transformed with pYES plasmid encoding IPV- or IPD-DSPP (inducible GAL promoter), selected on appropriate nutrient media, and selected colonies induced for 5 hr. Twenty percent of concentrated media (Amicon Ultra-4 Centrifugal Filter Units, 10 kDa cutoff Millipore Sigma) was used for western blot analysis. Anti-Flag mouse monoclonal (M2) was used as primary detection antibody. LI-COR IR-fluorescent anti-mouse second antibody was used for detection on LI-COR’s Odyssey scanner. DSPP, dentin sialophosphoprotein; ER, endoplasmic reticulum; ER-ESCAPE motif, ER-Exit by Soluble Cargo using Amino-terminal Peptide-Encoding motif; Erv29p, ER-derived vesicles protein 29; IPD, isoleucine-proline-aspartic acid; IPV, isoleucine-proline-valine; IR, infrared.
(TIF)

S2 Fig Generation of three independent HEK293A *Surf4*^KO^ cell lines by CRISPR/Cas9 technology. (A) *Surf4* locus on human chromosome 9 targeted by CRISPR/Cas9. The location of targeted sequences is shown underlined in green. (B) PCR genotyping of 11 selected clones. Wild-type Surf4 shows a 328 bp band, whereas the candidate KO Surf4 clones resulted the predicted smaller band, approximately 240 bp. (C) Sequence analysis of 3 selected *Surf4* KO clones. Cas9, CRISPR-associated 9; CRISPR, clustered regularly interspaced short palindromic repeat; HEK293A, human embryonic kidney cell line 293A; KO, knockout; Surf4, surfeit locus protein 4.
(TIF)

S3 Fig Sequence alignment of human Surf4 (NP_149351.1) and *S*. *cerevisiae* Erv29p (NP_011800.3) shows 30% identity by EMBOSS Needle Global Alignment program (https://www.ebi.ac.uk/Tools/psa/emboss_needle/). Erv29p, ER-derived vesicles protein; Surf4, surfeit locus protein 4.
(DOCX)

S4 Fig The rER and quality control domain lumenal proteins cyclophilin B and calreticulin of HEK293A cells were released predominantly by the NP-40 detergent, not by digitonin. Immunoblots of HEK293A proteins released by cholesterol-patch detergent, CEB (digitonin), or the rER/quality control membrane-solubilizing detergent NP-40. Five percent of cell extracts were used for western blot analyses with detection with mouse monoclonal primary antibody to calreticulin (FMC75, Calbiochem) and rabbit polyclonal antibody to cyclophilin B (Abcam). LI-COR IR-fluorescent second antibodies were used for detection on LI-COR’s Odyssey scanner. CEB, Cytosol Extraction Buffer; HEK293A, human embryonic kidney cell line 293A; IR, infrared; rER, rough endoplasmic reticulum.
(TIF)

S5 Fig Supporting Fig 5—Original microscopy image. (TIF)

S1 Table Accession number, species name, and brief surrounding sequence of representative species for each tripeptide from Fig 1. (XLSX)

S2 Table Accession number, species name, and brief surrounding sequence of representative species for each tripeptide from Fig 7. (XLSX)

S3 Table Examples of starting tripeptides of mating factor alpha-1 in 45 *Saccharomycetales*, including accession number, species name, and brief surrounding sequence. (XLSX)

S4 Table Yeast strains and genotypes used in this study. (XLSX)

S5 Table Yeast drop-out medium used in this study. (XLSX)

## Abbreviations

Φ-P-Φ: hydrophobic-proline-hydrophobic
Φ-S-Φ: hydrophobic-serine-hydrophobic
AAK: alanine-alanine-lysine
AMELX: amelogenin, X-linke
AMBN: ameloblastin
AMTN: amelotin
APA: alanine-proline-alanine
APD: alanine-proline-aspartic acid
API: alanine-proline-isoleucine
APT: alanine-proline-threonine
APV: alanine-proline-valine
AIA: alanine-isoleucine-alanine
BiP: binding immunoglobulin protein
BMP1: bone morphogenetic protein 1
BSP: bone sialoprotein
Cab45: 45 kDa Ca^2+^-binding protein
CALR: calreticulin
Cas9: CRISPR-associated 9
CEB: Cytosol Extraction Buffer
COL1A1: collagen type 1 alpha 1
COL2A1: collagen type 2 alpha 1
COL3A1: collagen type 3 alpha 1
COL6A1: collagen type 6 alpha 1
COL6A2: collagen type 6 alpha 2
COL6A3: collagen type 6 alpha 3
COL7A1: collagen type 7 alpha 1
COPII: coat protein complex II
CPY: carboxypeptidase Y
CRISPR: clustered regularly interspaced short palindromic repea
DD: dentin dysplasia
DGI: Dentinogenesis Imperfecta
DKR: aspartic acid-lysine-arginine
DMEM: Dulbecco's Modified Eagle Medium
DMP1: dentin matrix acidic phosphoprotein 1
DPP: dentin phosphoprotein
DSP: dentin sialoprotein
DSPP: dentin sialophosphoprotein
DYP: aspartic acid-tyrosine-proline
EDP: glutamic acid–aspartic acid–proline
EEE: glutamic acid–glutamic acid–glutamic acid
EEI: glutamic acid–glutamic acid–isoleucine
EET: glutamic acid-glutamic acid-threonine
EGT: glutamic acid-glycine-threonine
Emp24p: 24 kDa endomembrane protein
EMP46p: 46 kDa endomembrane protein
Emp47p: 47 kDa endomembrane protein
ENAM: enamelin
EPA: glutamic acid-proline-alanine
EPL: glutamic acid-proline-leucine
EPT: glutamic acid-proline-threonine
EPV: glutamic acid-proline-valine
ER: endoplasmic reticulum
ERAD: ER-associated degradation
ERES: ER exit site
ER-ESCAPE motif: ER-Exit by Soluble Cargo using Amino-terminal Peptide-Encoding motif
ERGIC: ER-Golgi intermediate compartment
ERO1: ER oxidoreductase 1
Erp1p: erythrocyte protein 1
Erp2p: erythrocyte protein 2
Erv14p: ER-derived vesicles protein 14
Erv15p: ER-derived vesicles protein 15
Erv25p: ER-derived vesicles protein 25
Erv26p: ER-derived vesicles protein 26
Erv29p: ER-derived vesicles protein 29
Erv46p: ER-derived vesicles protein 46
EST: glutamic acid-serine-threonine
F-GRP78: fungal GRP78
FGT: phenylalanine-glycine-threonine
FLT: phenylalanine-leucine-threonine
FPE: phenylalanine-proline-glutamic acid
FPM: phenylalanine-proline-methionine
FPR: phenylalanine-proline-arginine
FPT: phenylalanine-proline-threonine
FPV: phenylalanine-proline-valine
FQV: phenylalanine-glutamine-valine
FSA: phenylalanine-serine-alanine
FSM: phenylalanine-serine-methionine
FST: phenylalanine-threonine-valine
FTV: phenylalanine-threonine-valine
FVN: phenylalanine-valine-asparagine
GH: growth hormone
GPI: glycophosphatidylinositol
GPV: glycine-proline-valine
GRP78: glucose-related protein 78
GRP94: glucose-related protein 94
HA: hemagglutinin
HEK293A: human embryonic kidney cell line 293A
hGH: human growth hormone-1
HSV: histidine-serine-valine
IEV: isoleucine-glycine-valine
IGV: isoleucine-glycine-valine
IIP: isoleucine-isoleucine-proline
ILV: isoleucine-leucine-valine
INV: isoleucine-asparagine-valine
IPA: isoleucine-proline-alanine
IPD: isoleucine-proline-aspartic acid
IPE: isoleucine-proline-glutamic acid
IPP: isoleucine-proline-proline
IPR: isoleucine-proline-arginine
IPS: isoleucine-proline-serine
IPV: isoleucine-proline-valine
IRE1: inositol-requiring enzyme 1
IRV: isoleucine-arginine-valine
ISH: isoleucine-serine-histidine
ISL: isoleucine-serine-leucine
ISP: isoleucine-serine-proline
ISQ: isoleucine-serine-glutamine
ISR: isoleucine-serine-arginine
ISV: isoleucine-serine-valine
ITV: isoleucine-threonine-valine
KAV: lysine-alanine-valine
Kex2p: kexin 2
KGV: lysine-glycine-valine
KO: knockout
KSV: lysine-serine-valine
KVH: lysine-valine-histidine
LMAN1: lectin mannose-binding 1
LPO: lactoperoxidase
LPO-Gluc: LPO with carboxy-terminal luciferase
MCFD2: multiple coagulation factor deficiency protein 2
mDSP: mouse DSP
mfα-1: mating factor alpha-1
MPL: methionine-proline-leucine
NCBI: National Center for Biotechnology Information
NPV: asparagine-proline-valine
NSP: asparagine-serine-proline
NTT: asparagine-threonine-threonine
NUCB1: nucleobindin-1
OPN: osteopontin
PBS-T: PBS with 1% Tween 20
PDIA2: protein disulfide isomerase family A member 2
PDIA4: protein disulfide isomerase family A member 4
PrA: yeast aspartate protease
proMMP-9: pro-matrix metalloproteinase-9
PRY: pathogen-related yeast
PTH: parathyroid hormone
QC: quality control
QEE: glutamine-glutamic acid-glutamic acid
QPV: glutamine-proline-valine
QQQ: glutamine-glutamine-glutamine
QQV: glutamine-glutamine-valine
QSV: glutamine-serine-valine
QTT: glutamine-threonine-threonine
rER: rough ER
Rer1p: retention of ER proteins 1
RGV: arginine-glycine-valine
RLV: arginine-leucine-valine
RPA: arginine-proline-alanine
RPG: arginine-proline-glycine
RPK: arginine-proline-lysine
RPL: arginine-proline-leucine
RPV: arginine-proline-valine
RRR: arginine-arginine-arginine
RSV: arginine-serine-valine
SEAP: secreted alkaline phosphatase
SIBLING: small integrin-binding ligand, N-linked glycoprotein
siRNA: small interfering RNA
SLT: serine-leucine-threonine
SMT: serine-methionine-threonine
SPT: serine-proline-threonine
SPV: serine-proline-valine
SRT: serine-arginine-threonine
SST: serine-serine-threonine
Surf4: surfeit locus protein 4
TANGO1: transport and Golgi organization 1
TAI: threonine-alanine-isoleucine
TPV: threonine-proline-valine
VPA: valine-proline-alanine
XBP-1: X-box-binding protein 1
YPD: tyrosine-proline-aspartic acid
YPY: tyrosine-proline-tyrosine
