# Supplementary figures and images for: Surf4 (Erv29p) binds amino-terminal tripeptide motifs of soluble cargo proteins with different affinities, enabling prioritization of their exit from the endoplasmic reticulum
Source: PLoS Biol. 2018 Aug 7;16(8):e2005140. doi: 10.1371/journal.pbio.2005140 (PMC6097701; doi:10.1371/journal.pbio.2005140)

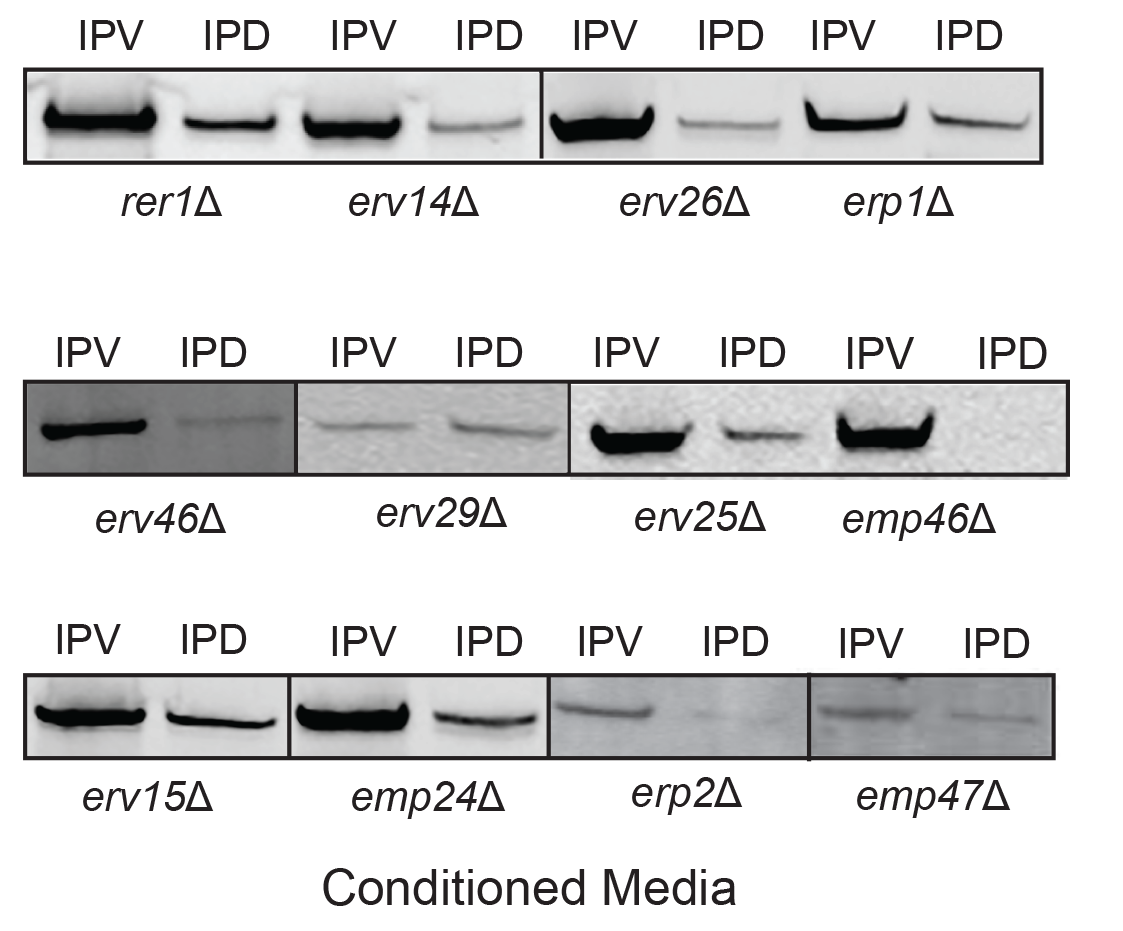

Supplement: S1 Fig — Immunoblot of wild-type DSPPFlag (IPV) or mutant (IPD) conditioned media in the indicated 12 ER exit vesicle-rich, transmembrane protein knockout strains. Cells were transformed with pYES plasmid encoding IPV- or IPD-DSPP (inducible GAL promoter), selected on appropriate nutrient media, and selected colonies induced for 5 hr. Twenty percent of concentrated media (Amicon Ultra-4 Centrifugal Filter Units, 10 kDa cutoff Millipore Sigma) was used for western blot analysis. Anti-Flag mouse monoclonal (M2) was used as primary detection antibody. LI-COR IR-fluorescent anti-mouse second antibody was used for detection on LI-COR’s Odyssey scanner. DSPP, dentin sialophosphoprotein; ER, endoplasmic reticulum; ER-ESCAPE motif, ER-Exit by Soluble Cargo using Amino-terminal Peptide-Encoding motif; Erv29p, ER-derived vesicles protein 29; IPD, isoleucine-proline-aspartic acid; IPV, isoleucine-proline-valine; IR, infrared. (TIF) [file pbio.2005140.s002.tif]

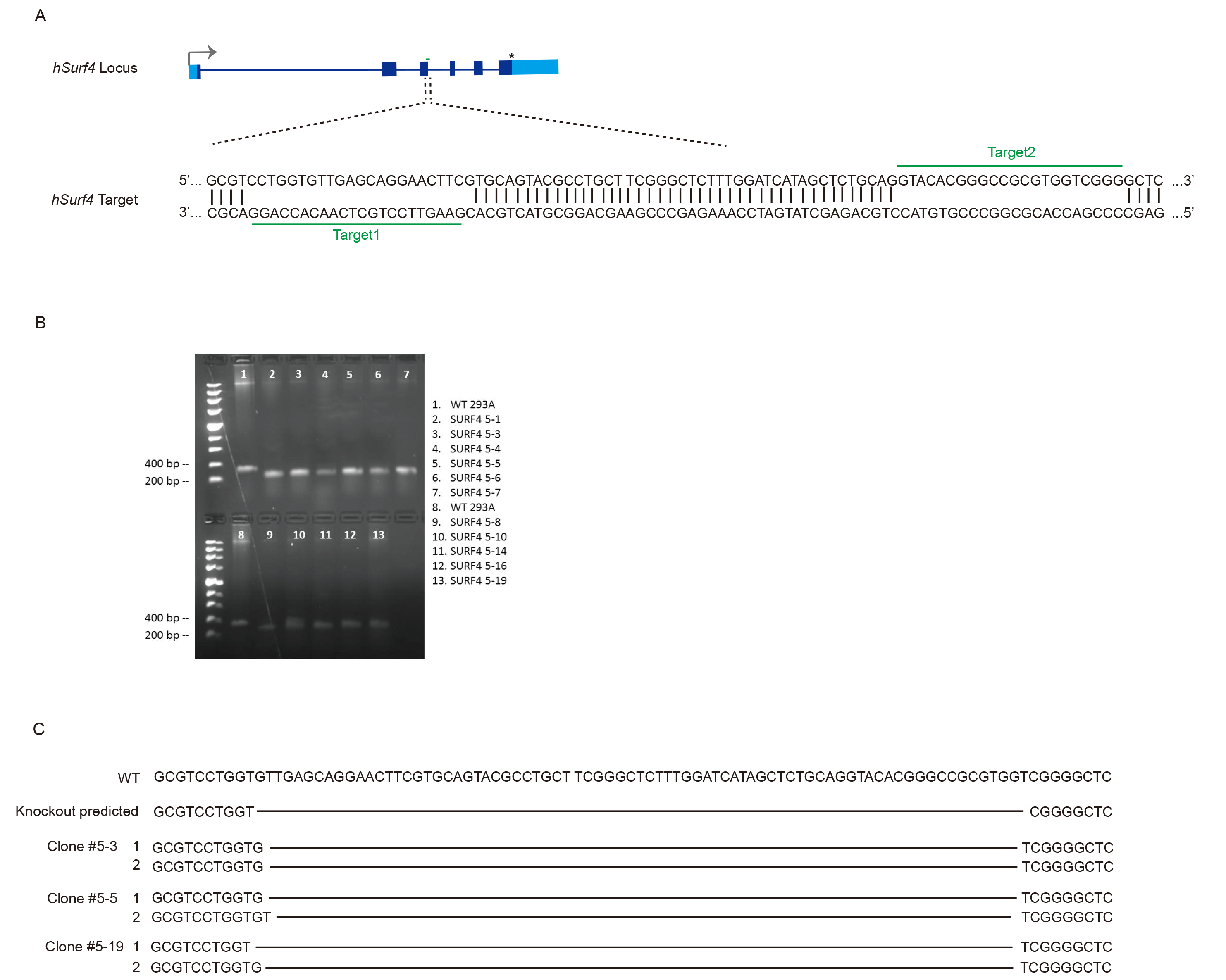

Supplement: S2 Fig — (A) Surf4 locus on human chromosome 9 targeted by CRISPR/Cas9. The location of targeted sequences is shown underlined in green. (B) PCR genotyping of 11 selected clones. Wild-type Surf4 shows a 328 bp band, whereas the candidate KO Surf4 clones resulted the predicted smaller band, approximately 240 bp. (C) Sequence analysis of 3 selected Surf4 KO clones. Cas9, CRISPR-associated 9; CRISPR, clustered regularly interspaced short palindromic repeat; HEK293A, human embryonic kidney cell line 293A; KO, knockout; Surf4, surfeit locus protein 4. (TIF) [file pbio.2005140.s003.tif]

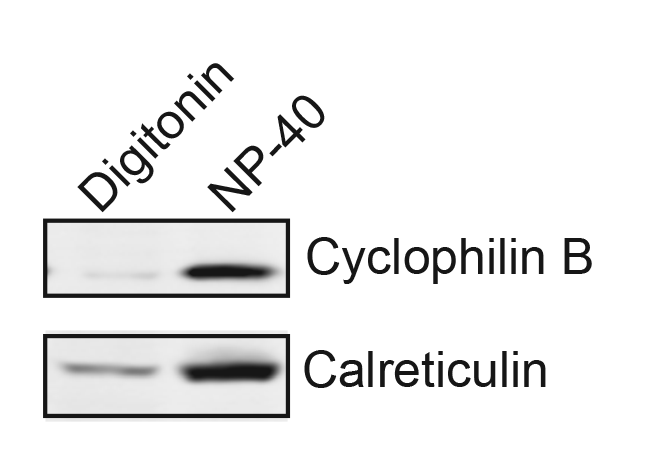

Supplement: S4 Fig — Immunoblots of HEK293A proteins released by cholesterol-patch detergent, CEB (digitonin), or the rER/quality control membrane-solubilizing detergent NP-40. Five percent of cell extracts were used for western blot analyses with detection with mouse monoclonal primary antibody to calreticulin (FMC75, Calbiochem) and rabbit polyclonal antibody to cyclophilin B (Abcam). LI-COR IR-fluorescent second antibodies were used for detection on LI-COR’s Odyssey scanner. CEB, Cytosol Extraction Buffer; HEK293A, human embryonic kidney cell line 293A; IR, infrared; rER, rough endoplasmic reticulum. (TIF) [file pbio.2005140.s005.tif]

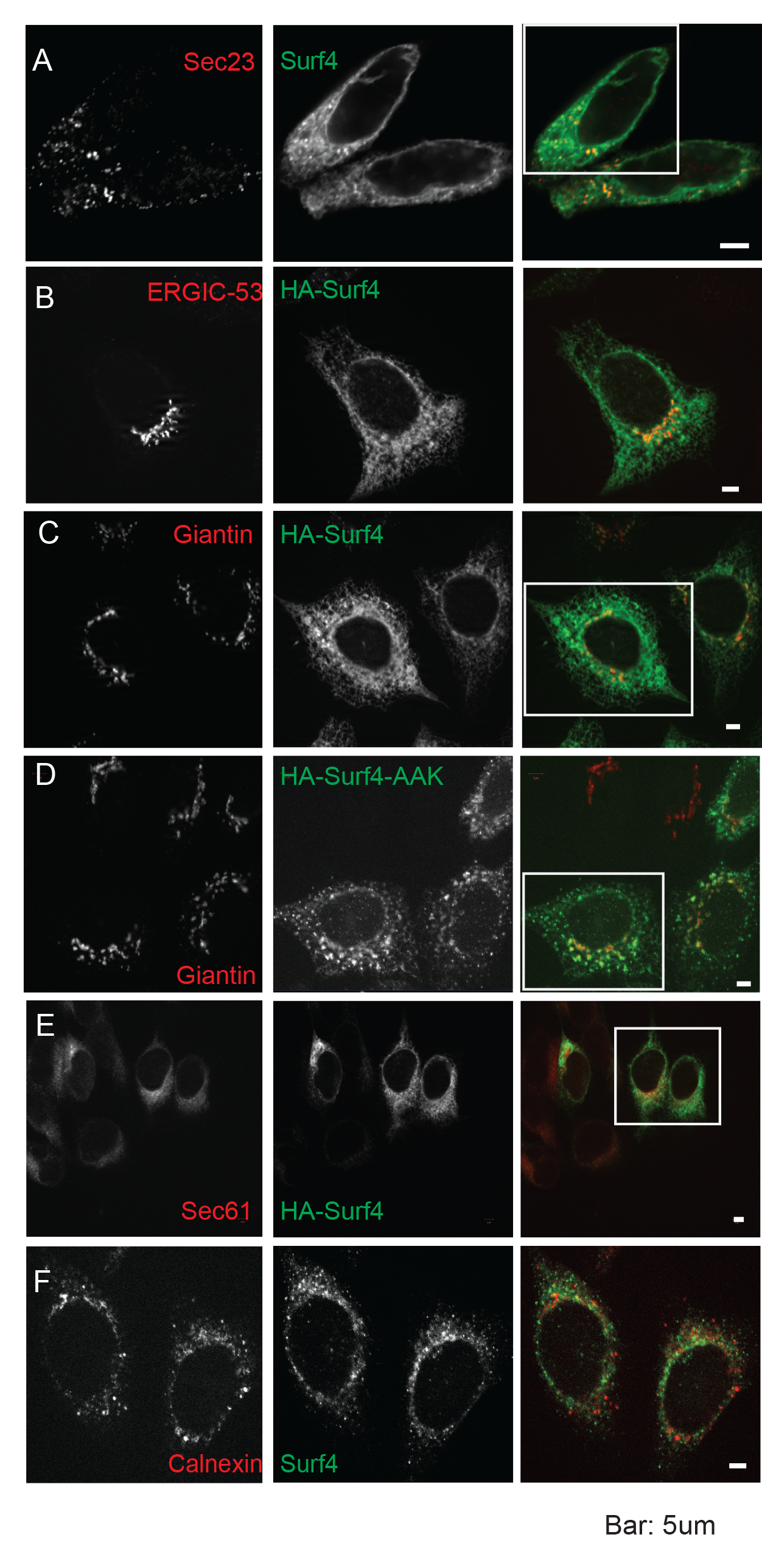

Supplement: S5 Fig — (TIF) [file pbio.2005140.s006.tif]
