# Supplementary material for: Surf4 (Erv29p) binds amino-terminal tripeptide motifs of soluble cargo proteins with different affinities, enabling prioritization of their exit from the endoplasmic reticulum
Source: PLoS Biol. 2018 Aug 7;16(8):e2005140. doi: 10.1371/journal.pbio.2005140 (PMC6097701; doi:10.1371/journal.pbio.2005140)
Supplement: S3 Fig — Erv29p, ER-derived vesicles protein; Surf4, surfeit locus protein 4. (DOCX) [file pbio.2005140.s004.docx]

S3 Fig

SURF4_HUMAN 1 -----------------------------MGQNDLMG--------TAEDF 13

..||.... :.|.|

ERV29_YEAST 1 MSYRGPIGNFGGMPMSSSQGPYSGGAQFRSNQNQSTSGILKQWKHSFEKF 50

SURF4_HUMAN 14 ADQFLRVT--------KQYLPHVARLCLISTFLEDGIRMWFQWSEQRDYI 55

|.:...:| |.|:|.::|..:::||.||..|:..|||:|..|:

ERV29_YEAST 51 ASRIEGLTDNAVVYKLKPYIPSLSRFFIVATFYEDSFRILSQWSDQIFYL 100

SURF4_HUMAN 56 DTTWNCGYLLASSFVFLNLLGQLTGCVLVLSRNFVQYACFGLFGIIALQT 105

:...:..|.....|:.:..:..|.|..|::.|....||...|...:..|.

ERV29_YEAST 101 NKWKHYPYFFVVVFLVVVTVSMLIGASLLVLRKQTNYATGVLCACVISQA 150

SURF4_HUMAN 106 IAYSILWDLKFLMRNLALGGGLLLLLAESRSEGKSMFAGVPTM--RESSP 153

:.|.:.....|::||.::.||||:..::|..:.|:.|..:|.: :....

ERV29_YEAST 151 LVYGLFTGSSFVLRNFSVIGGLLIAFSDSIVQNKTTFGMLPELNSKNDKA 200

SURF4_HUMAN 154 KQYMQLGGRVLLVLMFMTLLHFDASFFSIVQNIVGTALMILVAIGFKTKL 203

|.|:...||:|:||||:... |..|:|::|..|:|| |..|||:|||.

ERV29_YEAST 201 KGYLLFAGRILIVLMFIAFT-FSKSWFTVVLTIIGT---ICFAIGYKTKF 246

SURF4_HUMAN 204 AALTLVVWLFAINVYFNAFWTIPVYKPMHDFLKYDFFQTMSVIGGLLLVV 253

|::.|.:.|...|:..|.:|.....| .|||||:|:|.:|:|||||||.

ERV29_YEAST 247 ASIMLGLILTFYNITLNNYWFYNNTK--RDFLKYEFYQNLSIIGGLLLVT 294

SURF4_HUMAN 254 ALGPGGVSMDEKKKEW 269

..|.|.:|:|||||.:

ERV29_YEAST 295 NTGAGELSVDEKKKIY 310

Length: 316

Identity: 95/316 (30.1%)

Similarity: 150/316 (47.5%)
